# Effects of feeding strategies on culture performance and product quality in NISTCHO

**DOI:** 10.1038/s41540-026-00686-3

**Published:** 2026-03-19

**Authors:** Veronika Schäpertöns, Larissa Hofer, Thomas Berger, Jerneja Štor, Nicolas Marx, Wolfgang Esser-Skala, Thomas Rauter, Dominik Hofreither, Laura Liesinger, Peter Filzmoser, Ruth Birner-Gruenberger, Christian G. Huber, Nicole Borth, Nikolaus Fortelny

**Affiliations:** 1 Department of Biosciences and Medical Biology, University of Salzburg, Salzburg, Austria; 2 Department of Biotechnology and Food Science, Institute of Animal Cell Technology and Systems Biology, BOKU University, Vienna, Austria; 3 Faculty of Technical Chemistry, Institute of Chemical Technologies and Analytics, TU Wien, Vienna, Austria; 4 Institute of Statistics and Mathematical Methods in Economics, TU Wien, Vienna, Austria; 5 Center for Tumor Biology and Immunology (CTBI), University of Salzburg, Salzburg, Austria

**Keywords:** Biochemistry, Biological techniques, Biotechnology

## Abstract

Monoclonal antibody *N*-glycosylation is a critical quality attribute influencing therapeutic safety and efficacy, and is strongly influenced by bioprocess design. NISTCHO, a publicly available Chinese hamster ovary producer cell line, is increasingly encouraged for use as a reference system. However, the impact of feeding strategies on cellular performance and *N*-glycosylation has not been assessed. Here, we applied multivariate analysis of compositional *N*-glycan data to assess how feeding strategies influence *N*-glycan composition of cNISTmAb. We varied feeding strategies in frequency, glucose supply, and galactose/manganese supplementation. Feeding frequency had minimal impact on quality attributes but strongly affected culture performance, with every-other-day feeding improving titers and cell-specific productivity. High glucose availability supported growth and productivity. Low glucose strategies reduced titers and shifted *N*-glycosylation towards non-galactosylated and fucosylated species, despite lactate accumulation remaining within favorable ranges. Galactose and manganese consistently increased antibody galactosylation, with galactose additionally serving as an auxiliary carbon source, extending cell viability. Importantly, mAb glycation remained stable across all feeding strategies at harvest. Overall, these results demonstrate that feed composition and timing can be used to tune both cellular performance and mAb glycosylation, establishing NISTCHO as a robust benchmark for standardized process-quality studies.

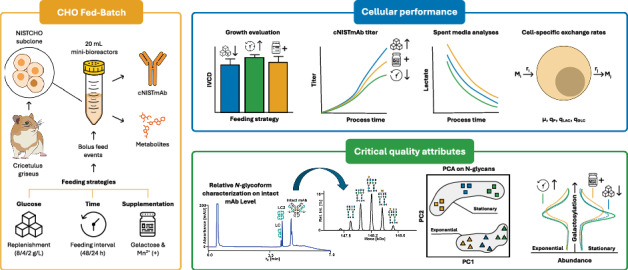

## Introduction

Monoclonal antibodies (mAbs) are a rapidly growing area in the market of therapeutic recombinant proteins, enabling targeted treatments for various diseases, including cancer and autoimmune disorders. In 2021, mAbs accounted for a market value of $217 billion and represented 15 out of the 20 top-selling biopharmaceutical products^1^. The Chinese hamster ovary (CHO) cell line is the preferred platform for producing mAbs, especially because it produces mAbs with human-like post-translational modifications^1^.

To drive innovation and collaboration of the biopharmaceutical academic and industrial community, a new mammalian reference cell line called NISTCHO^2^ was released by NIST (National Institute of Standards and Technology) in 2023. Due to its relevance as a central reference resource, this cell line is rapidly being adopted by the community. The first report of the development and the initial characterization of NISTCHO was carried out by Dahodwala et al.^3^. NISTCHO expresses a non-originator, humanized IgG1*κ* mAb known as cNISTmAb, which exhibits a characteristic *N*-glycan profile of CHO cells, but the relative *N*-glycan abundance differs from the murine suspension culture-derived originator^2–4^. Recent community efforts that established NISTCHO as a reference cell line were reported by Fratz-Berilla et al., who developed and optimized seed train, fed-batch, and perfusion bioreactor processes with an emphasis on platform establishment^5^. In summary, NISTCHO is now ripe to serve as a platform for standardized bioprocess optimization.

The availability of amino acids, vitamins, salts, and trace elements in the medium is a key determinant in cellular metabolism and directly impacts culture physiology and critical quality attributes (CQAs)^6,7^. Moreover, the concentration and frequency at which nutrients are delivered to the cells, as dictated by the feeding strategy, can affect productivity and glycosylation patterns. Ideal bioprocess conditions maximize cell-specific productivity and sustain growth rates, while minimizing by-product formation (e.g., lactate)^6,7^. Various studies have shown that media additives such as galactose and typically manganese can positively modulate galactosylation by acting as precursors or cofactors in glycan biosynthesis pathways^8–14^. Systematic control of nutrient supply and supplementation strategies, therefore, represents a powerful and cost-effective approach to steer cellular productivity and CQAs, without relying on genetic engineering^15^. Unfortunately, due to the diversity in cell line and subclone behavior, such strategies have to be optimized anew for each new production process.

NISTCHO is a useful testcase to investigate effects of critical process parameters (CPPs) on CQAs, where especially *N*-glycosylation plays a central role in determining pharmacokinetics, stability, and efficacy^16^. The structure of *N*-glycans attached to the conserved Fc domain modulates key immune effector functions, including antibody-dependent cellular cytotoxicity (ADCC) and complement-dependent cytotoxicity (CDC)^17^. Notably, an increased abundance of terminal *β*1,4-linked galactose residues has been associated with enhanced CDC activity^18,19^, whereas the absence of a core *α*1,6-linked fucose enhanced ADCC^20,21^. Understanding the factors that influence the composition of these glycan structures is especially important for the development of biosimilars^1^.

One important factor is the availability of glucose, which is metabolized into nucleotide sugar donors, necessary for *N*-glycosylation. Glucose depletion during cell culture has been shown to reduce galactosylation and sialylation levels of *N*-glycans, particularly when depletion persists over time^22,23^. In addition to *N*-glycosylation, high glucose levels in the culture medium can also increase glycation of the mAb itself (not of the *N*-glycans)—a non-enzymatic reaction occurring extracellularly after antibody secretion into the medium^24^. Although mAb glycation is less frequently discussed in the literature, it can also negatively affect therapeutic quality^25^. Importantly, it is difficult to analytically distinguish mAb glycation from mAb *N*-glycosylation on an intact protein level because glucose and galactose have the same masses (162 Da). This results in a so-called hexosylation bias in glycoprofiling on an intact level^26^. To address this bias, mAb glycation measured on mAb without *N*-glycans can be used as a quality attribute and to correct the hexosylation bias^26^.

Few systematic studies have investigated how specific feeding strategies, such as (1) variations in glucose availability, (2) changes in feeding frequency, and (3) the supplementation of galactose and manganese, jointly influence cellular performance and CQAs. In particular, for the NISTCHO cell line and its product cNISTmAb, there is yet limited data due to the novelty of this open-access reference cell line. Here, we systematically analyze how feeding strategies affect process characteristics, cell-specific rates, as well as CQAs. We developed a dedicated computational pipeline that uses clr transformation of the *N*–glycan abundances to enable analyses across different *N*-glycans, similarly to transcriptomics and other -omics. Through integration of bioprocess data and extensive glycan profiling at both exponential and stationary phases, we establish how upstream process control can influence downstream product quality in a reproducible system.

## Results

### Carbon source availability determines process characteristics

**Fig. 1:**
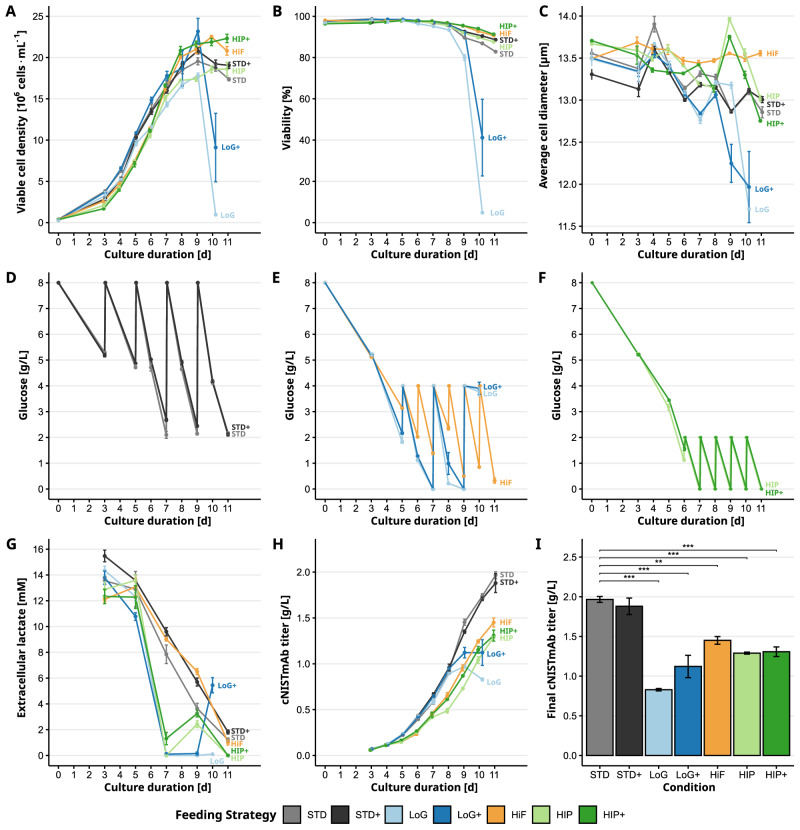
Effects of feeding strategies on process characteristics. **A** Viable cell density (VCD), **B** viability, **C** average cell diameter. Extracellular glucose concentrations for feeding strategies **D** STD and STD+, **E** LoG, LoG+ and HiF and **F** HIP and HIP+. **G** Extracellular lactate concentrations, **H** accumulation of cNISTmAb titer, and **I** final cNISTmAb titers. Color code: STD (Standard) in gray, STD+ (Standard+) in black, LoG (Low Glucose) in light blue, LoG+ (Low Glucose+) in dark blue, HiF (Higher frequency) in orange, HIP (HIPDOG-like) in light green, HIP+ (HIPDOG-like+) in darkgreen. Statistical analyses show pairwise comparisons of all strategies to the STD strategy. Adjusted *p*-values were used to assign significance levels, with thresholds defined as *p* < 0.05 (*), *p* < 0.01 (**), and *p* < 0.001 (***). Error bars represent the standard error of the biological replicates.

We investigated seven fed-batch feeding strategies (Table 1) to assess their impact on NISTCHO cell growth and productivity. Four strategies differed in glucose concentration per feed (2.0, 4.0, or 8.0 g/L), two variations in the feeding interval (daily vs. every-other-day), and selected strategies were supplemented with Gal+. Gal+ contains galactose, which can serve both as an alternative carbon source and a precursor for UDP-galactose, as well as manganese, a trace element that functions as a cofactor in *N*-glycosylation. These combinations allowed us to isolate the effects of glucose availability, feeding frequency, and Gal+ supplementation on process performance. The different feeding strategies led to distinct process profiles, as reflected by growth dynamics (viable cell density, viability, cell diameter), extracellular metabolite concentrations (glucose and lactate), and monoclonal antibody (cNISTmAb) production, as shown in Fig. 1. Up to day 9, all strategies displayed similar growth dynamics. Thereafter, the low glucose variants show a rapid decline in viable cell numbers and viability, due to carbon source depletion (Fig. 1A). Maximum VCDs ranged from 17.0 to 26.0 million cells/mL (HIP and LoG+, respectively).

**Table 1:** Implemented feeding strategies based on varying intervals and concentrations of feeds and additives

Abbrev.	Feed interval [h]	Glucose replenishment [g/L]	Gal+ supplement	Description
STD	48	8	No	Standard method
STD+	48	8	Yes	STD with Gal+
LoG	48	4	No	Low glucose
LoG+	48	4	Yes	LoG with Gal+
HiF	24	4	No	Higher frequency feeding
HIP	24	2	No	HIPDOG-like feeding
HIP+	24	2	Yes	HIP with Gal+

Cell viability (Fig. 1B) remained above 90% across all feeding strategies, with the exception of the LoG and LoG+ conditions, which exhibited a pronounced decline in viability starting at day 8 and 9, respectively. By day 10, cultures under the LoG strategy showed a marked decline in viability across all replicates, reaching approximately 8%, indicative of process collapse. Meanwhile, the LoG+ strategy, supplemented with an additional carbon source (galactose), maintained viability for approximately one additional day and exhibited greater variability at day 10 as indicated by a higher standard error of the mean. All other feeding strategies sustained high viability over a longer cultivation period with an average of 90%.

Average cell diameter only changed moderately across the different feeding strategies, with a range from 13 to 14 μm (Fig. 1C). Most strategies showed a slight decrease in diameter toward the end of cultivation, likely due to substrate limitations, a behavior that is more prominently observed in batch cultures. Another reason could be the measurement interference of dead and apoptotic cells. This is supported by the fact that HiF cells showed a very consistent cell size throughout the bioprocess, while both LoG and LoG+ exhibited a sharp decline in average diameter.

Glucose dynamics under the various feeding strategies are shown in Fig. 1D, E, F. We observed that in cultures replenished to 8.0 g/L (STD and STD+), glucose concentrations remained elevated and never dropped below 2.0 g/L (11.1 mM). When less glucose was fed (LoG and LoG+; 4.0 g/L every other day), glucose depletion occurred earlier, with concentrations dropping below 1.0 g/L by day 6 and reaching complete depletion by day 7. Cells continued to metabolize lactate (and galactose, if supplemented) until both carbon sources were exhausted at day 9, resulting in culture collapse. Increasing feeding frequency to daily (HiF, 4.0 g/L) prevented full depletion, though glucose fell to 0.5 g/L after day 9. The HIP and HIP+ conditions, with minimal glucose replenishment (2.0 g/L daily), exhibited cyclic depletion patterns but maintained high VCD and viability during the stationary phase. However, lower final mAb titers (Fig. 1I) compared to STD and STD+ strategies highlight that insufficient glucose levels limit recombinant protein production.

The feeding strategies induced distinct metabolic and productivity profiles, largely driven by differences in energy source availability. Extracellular lactate levels (Fig. 1G) revealed a metabolic shift in all strategies from production during early growth to consumption. This shift was most pronounced in glucose-limited strategies (LoG, HIP, and HIP+), where lactate was nearly depleted by day 7. In HIP and HIP+, a small secondary lactate spike at day 9 suggested transient reactivation of glycolysis when glucose was briefly available. This cyclic pattern of glucose depletion and reaccumulation observed in the HIP and HIP+ strategies is consistent with the metabolic control loop described for the HIPDOG system by Gagnon et al.^27^. When glucose levels reach below 1 mM, the cells take up lactic acid from the culture medium again, which increases the culture pH^27^. This rise in pH is a reliable surrogate marker of glucose limitation and was used here as the manual trigger for glucose addition. In our experiments, pH for HIP and HIP+ strategies consistently increased to approximately 7.4 from day 6 onward before each feeding event. After feeding, the cells again transiently produced lactate and lowered the pH until glucose became limited and the cycle restarted. Figure 1H shows the accumulation of cNISTmAb titer over time. Cultures with continuous glucose supply (STD and STD+) showed steep and sustained mAb accumulation from day 6 to 10. In contrast, LoG and LoG+ plateaued early, consistent with culture decline and energy source exhaustion. The decrease in titer at day 10 is most likely due to dilution accounted for by feeding and potentially the presence of extracellular proteases from cell lysis. The highest final titers (Fig. 1I) were seen with STD and STD+ strategies (>1.9 g/L), and the lowest in LoG (<0.9 g/L). Intermediate values were observed for LoG+, HiF, HIP, and HIP+ (1.1–1.5 g/L). All pairwise statistical comparisons for the final titers can be seen in Supplementary Fig. 2.

In summary, the availability of carbon sources-glucose and lactate-was a key determinant of growth, viability, and productivity. Strategies that failed to maintain adequate energy supply throughout the process, especially low glucose feeding, showed early growth arrest and reduced recombinant protein titers.

### Excess glucose ensures optimal growth and productivity rates

**Fig. 2:**
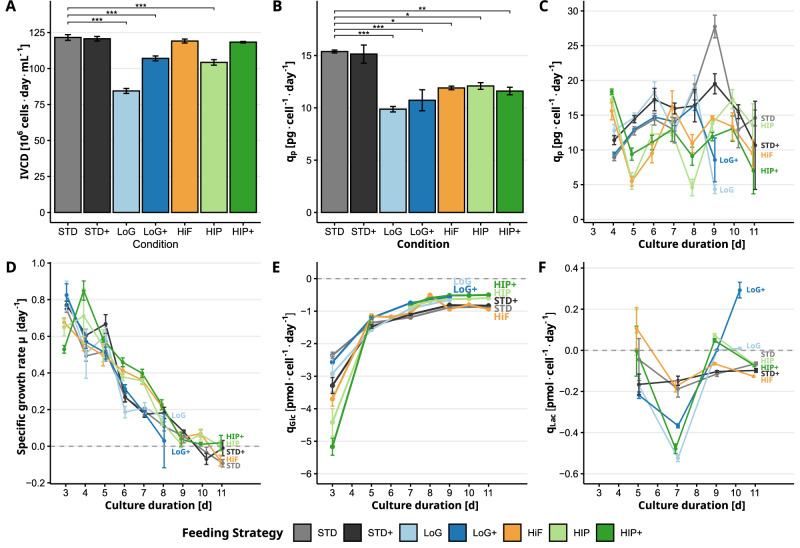
Effects of feeding strategies on IVCD and cell-specific rates. **A** Integral viable cell density (IVCD), **B** average cell-specific productivity (*q*_P_) of cNISTmAb across the entire duration, **C** *q*_P_ over time, **D** specific growth rate (*μ*) over time (the last timepoints of LoG and LoG+ strategies were removed to allow better visibility) **E** specific glucose consumption rate (*q*_GLC_) over time, and **F** specific lactate rate (*q*_LAC_) over time. Color code: STD (Standard) in gray, STD+ (Standard+) in black, LoG (Low Glucose) in light blue, LoG+ (Low Glucose+) in dark blue, HiF (Higher frequency) in orange, HIP (HIPDOG-like) in light green, HIP+ (HIPDOG-like+) in darkgreen. Statistical analyses show pairwise comparisons of all strategies to the STD strategy. Adjusted *p*-values were used to assign significance levels, with thresholds defined as *p* < 0.05 (*), *p* < 0.01 (**), and *p* < 0.001 (***). Error bars represent the standard error of the biological replicates.

To investigate the impact of the feeding strategies on cell growth and metabolism, we analyzed cell-specific rates over the course of the culture period (Fig. 2).

Integral viable cell density (IVCD) was used as a cumulative measure of cell growth over time (Fig. 2A). Compared to the STD strategy, the LoG and LoG+ and HIP strategies exhibited significantly reduced cell growth. In contrast, no notable differences in IVCD were observed for the STD+, HiF, and HIP+ strategies. The addition of Gal+ thus did not impact the growth of cultures with excess glucose, such as in the STD strategy. However, in glucose-limited strategies (LoG and HIP), galactose supplementation supported improved cell growth. All pairwise statistical observations can be seen in (Supplementary Fig. 1).

The average cell-specific productivity (*q*_P_) of cNISTmAb was calculated over the entire process duration from day 3 to 11 (Fig. 2B), as well as resolved over time (Fig. 2C). Notably, the STD and STD+ strategies achieved the highest average (${q}_{{{\rm{P}}}_{i}}$) values, significantly surpassing those observed under LoG and LoG+, HiF, and HIP and HIP+ strategies. In contrast to the IVCD (Fig. 2A), Gal+ supplementation did not enhance productivity under any strategy (see Supplementary Fig. 3). Temporal profiling of (${q}_{{{\rm{P}}}_{i}}$) revealed similar trends across all feeding strategies. Negative (${q}_{{{\rm{P}}}_{i}}$) values were observed for the LoG and LoG+ strategies, likely due to dilution effects following feed addition. This reduction in product concentration is consistent with the decline in cNISTmAb titers observed after day 9 (Fig. 1H).

Specific growth rates *μ* were used to assess proliferation dynamics over time (Fig. 2D). All strategies showed similarly high *μ* until day 5. Between day 6 and 8, daily feeding strategies exhibited higher growth rates compared to those fed every other day. This distinction disappeared after day 9, when all cultures entered the stationary phase and *μ* neared zero.

The *q*_GLC_ was used to assess changes in metabolic activity over time (Fig. 2E). The starting time for *q*_GLC_ calculations depended on the first feeding interval (as can be seen in Fig. 1D, E, F). All strategies showed high *q*_GLC_ consumption in the exponential growth phase, illustrating high reliance on glucose as the primary energy source. A lower *q*_GLC_ was observed for strategies with reduced glucose replenishment (LoG and LoG+, HIP and HIP+), which became apparent at day 7 until the end of the process. Strategies STD and STD+ and HiF consumed approximately 60.5% more glucose in this window.

The shift from glycolytic to more oxidative metabolism in the stationary phase was also reflected in the *q*_LAC_ time course (Fig. 2F). At day 5, some strategies were still producing lactate, while others had already shifted to consumption. The trend of lower glucose availability leading to reduced *q*_GLC_ (Fig. 2E) was mirrored by increased lactate metabolization. Especially at day 7, we observed a stronger peak lactate consumption for the LoG and LoG+ and HIP and HIP+ strategies. Brief lactate production phases in HIP and HIP+ indicate occasional excess glucose, driving temporarily positive *q*_LAC_.

Together, these cell-specific rates highlight distinct metabolic and growth responses to the applied feeding strategies. While only the glucose-limited strategies LoG and LoG+ and HIP showed reduced proliferation (Fig. 2A), all other strategies, except STD and STD+, exhibited impaired productivity (Fig. 2B). This highlights that the STD feeding strategy performed the best in terms of growth and productivity. The presence of excess glucose in the STD and STD+ and HiF strategies strongly influenced glucose and lactate metabolism. Notably, the benefits of Gal+ supplementation on growth were only observed under nutrient-limited strategies, where galactose could serve as an effective alternative carbon source. Among all employed feeding variations, the STD strategy consistently supported both robust cell growth and the highest productivities, highlighting its suitability for optimal bioprocess performance.

### Galactose supplementation and growth phase affect *N*-glycan heterogeneity

**Fig. 3:**
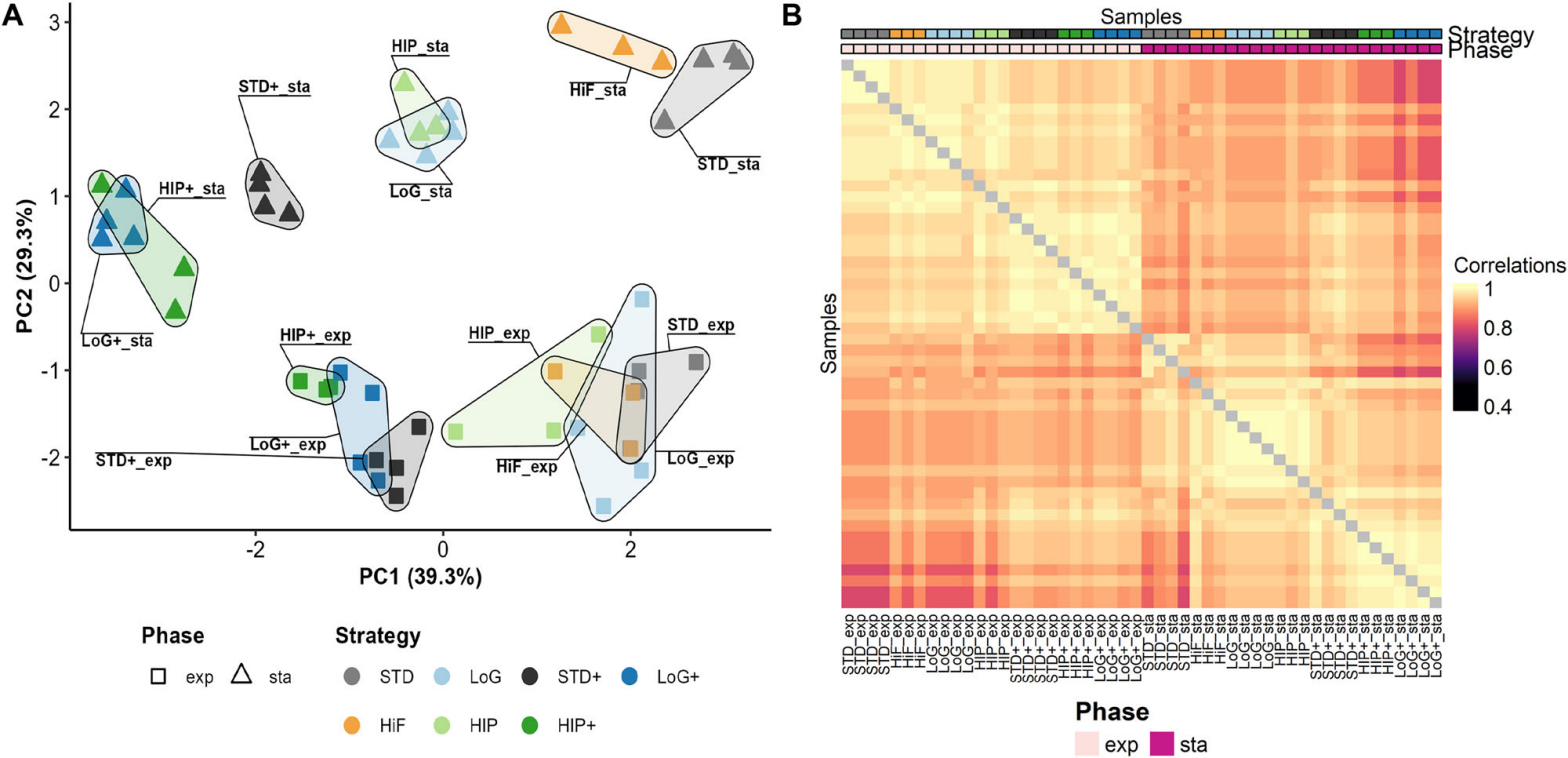
Data exploration of clr-transformed *N*-glycan data for all biological replicates and all bioprocess phases. **A** Principal component analysis for all samples and **B** sample correlations based on *N*-glycan abundance profiles. A sample-by-sample Spearman correlation matrix was computed across all quantified glycoforms (across all glycoforms), and the heatmap displays pairwise Spearman correlation coefficients between samples, as is common for omics data^53^. The diagonal squares contain autocorrelations of each sample with itself (correlation = 1), shown in gray to de-emphasize them. Row names were omitted to reduce redundancy and improve readability. The ordering of samples is identical on both axes. Color code: STD (Standard) in gray, STD+ (Standard+) in black, LoG (Low Glucose) in light blue, LoG+ (Low Glucose+) in dark blue, HiF (Higher frequency) in orange, HIP (HIPDOG-like) in light green, HIP+ (HIPDOG-like+) in darkgreen. Note: Legend for Feeding Strategy is the same for (**A**, **B**).

To investigate the variability of *N*-glycans across culture time points and feeding strategies, we first transformed fractional abundances to centered log-ratio (clr) values. This changes values from a constrained space (from zero to one) to an unconstrained space and thereby accounts for their compositional nature^28–30^. This, in turn, enabled us to perform principal component analysis (PCA) and pairwise correlation analysis to explore the (dis-)similarity of samples across feeding strategies and time points (Fig. 3).

PCA revealed (Fig. 3A) that samples collected during the exponential phase (exp) clustered more tightly than those collected during the stationary phase (sta), indicating a lower variability in glycan profiles across feeding strategies during the exponential growth phase. This observation was further supported by higher Spearman correlation coefficients among samples during the exponential phase (Fig. 3B).

Samples collected at the earlier time point (exp) clustered clearly based on presence or absence of Gal+ supplementation, suggesting a consistent glycosylation response to Gal+ addition (Fig. 3A). In contrast, at the later time point (sta), sample clustering became more heterogeneous, with distinct feeding strategies leading to more clearly discernable glycan profiles (Fig. 3B), while still maintaining the separation based on Gal+ supplementation. Nevertheless, HiF_sta and STD_sta showed close proximity, suggesting that daily and alternate-day feeding schedules with a comparable final glucose concentration resulted in similar *N*-glycan patterns. Likewise, HIP_sta and LoG_sta overlapped, indicating comparable effects of both strategies on glycan composition, likely due to depletion of glucose before the start of the next feeding interval (Fig. 1D–F).

To statistically test these observations, we performed PERMANOVA on clr-transformed *N*-glycan abundances. Culture phase and feeding strategy together explained 79.7% of the variation in glycan composition (*R*^2^ = 0.79, *p* = 0.001), and including the phase × strategy interaction increased explained variance to 90.4% (*R*^2^ = 0.90, *p* = 0.001), indicating that the effect of feeding strategy depended on growth phase. No difference among feeding strategies could be proven to be driven by heterogeneous dispersion (*p* = 0.11), whereas dispersion differed significantly between phases (*p* = 2.44 × 10^−6^), with stationary-phase samples exhibiting greater heterogeneity. Visual patterns observed in PCA and sample-to-sample correlations suggested differences in glycan profiles between phases and across feeding strategies. These patterns were statistically tested by PERMANOVA and dispersion analyses, confirming that both culture phase and feeding strategy significantly shape *N*-glycan variability. Importantly, the Gal+ supplementation was the main driver for heterogeneity of *N*-glycans, despite having little to no effect on the bioprocess characteristics (see Results Carbon source availability determines process characteristics).

### Fucosylation increases in stationary phase independent of feeding strategy

**Fig. 4:**
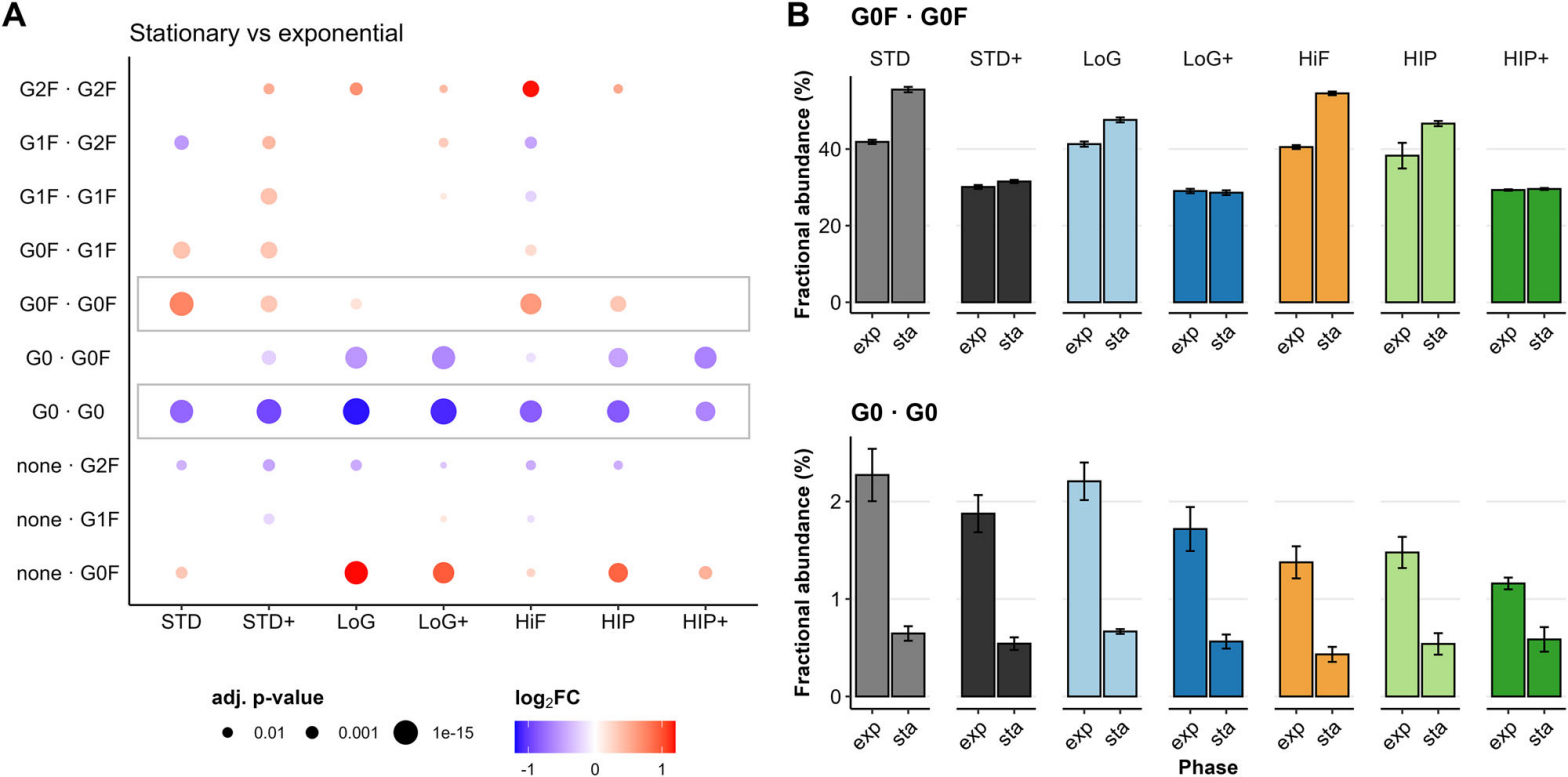
The impact of the collection time point on *N*-glycan abundances. **A** Dotplots show the log_2_FC and the adjusted *p*-values for a single feeding strategy, comparing the stationary time point against the exponential time point. Positive log_2_FC values mean higher abundance in the stationary phase, while negative log_2_FC values mean higher abundance in the exponential phase. **B** The fractional abundances of the glycoforms G0F ⋅ G0F (top) and G0 ⋅ G0 (bottom) as highlighted in (**A**) are shown for all feeding strategies. Color code: STD (Standard) in gray, STD+ (Standard+) in black, LoG (Low Glucose) in light blue, LoG+ (Low Glucose+) in dark blue, HiF (Higher frequency) in orange, HIP (HIPDOG-like) in light green, HIP+ (HIPDOG-like+) in darkgreen. Error bars represent the standard deviation of the biological replicates. of fractional abundances. For *N*-glycan nomenclature, please refer to Supplementary Fig. 5.

To assess the impact of culture phase and state on specific *N*-glycans attached to cNISTmAb, we systematically compared the clr-transformed relative abundances of individual glycoforms between the exponential and stationary phases within each feeding strategy. In every strategy tested, significant differences (adj. *p*-value < 0.05) in *N*-glycan abundances were observed (Fig. 4A), highlighting a strong influence of the time in culture on glycosylation profiles.

The magnitude and direction of these changes varied across strategies, indicating a high degree of heterogeneity in the temporal regulation of *N*-glycosylation. Among the most consistent trends, the G0F ⋅ G0F glycoform showed an increase during the stationary phase in the STD, HiF, and, to a lesser extent, in the STD+ and HIP strategies (Fig. 4B, G0F ⋅ G0F). In contrast, the afucosylated G0 ⋅ G0 glycoform consistently decreased at the stationary time points across all strategies (Fig. 4B, G0 ⋅ G0).

Interestingly, culture phase also affected galactosylation levels (Fig. 4A, G1F ⋅ G1F, G1F ⋅ G2F, G2F ⋅ G2F), particularly in the STD+ and LoG+ strategies, where galactosylated glycoforms were more abundant in the stationary phase. This increase was not observed in the HIP+ strategy, where no significant differences were detected for galactosylated glycoforms between time points (see Discussion).

In summary, the culture duration significantly influenced *N*-glycan composition with a specific galactosylation increase in Gal+ strategies (except for HIP+, see Discussion), a decrease in afucosylated species in all strategies, and an increase in fucosylated and non-galactosylated species in the STD and HiF strategies. These effects likely arise due to a decreased availability or utilization of glycosylation precursors in all observed feeding strategies.

### Availability of glucose and galactose determine antibody glycation and galactosylation

**Fig. 5:**
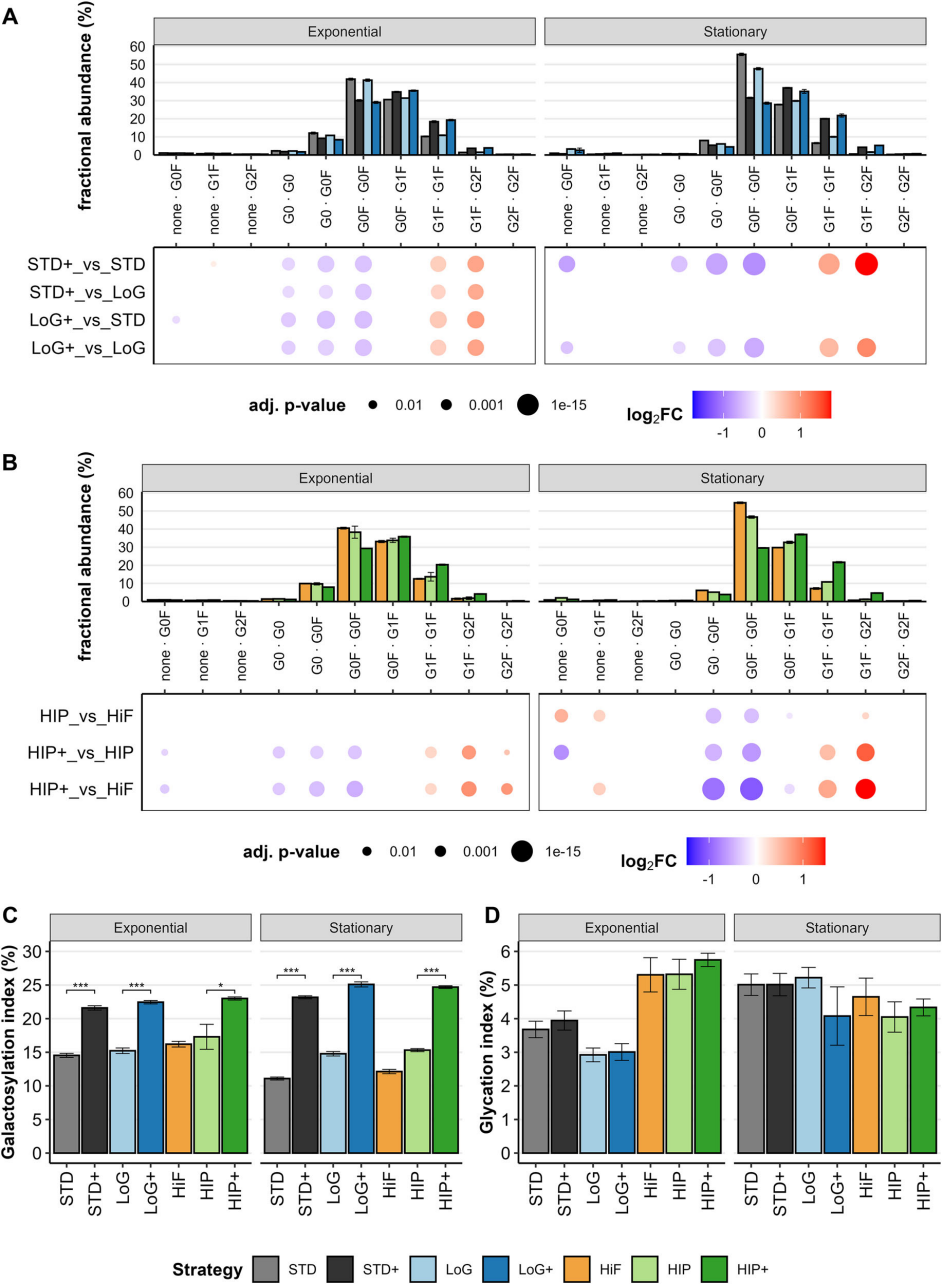
The impact of the feeding strategy on *N*-glycan abundances. **A** Fractional abundances of glycoforms for feeding strategies STD and STD+ and LoG and LoG+ (top). Dotplots show the log_2_FC and the adjusted *p*-values for two strategies at a given time point (bottom). **B** Fractional abundances of glycoforms for feeding strategies HiF, HIP, and HIP+ (top). Dotplots show the log_2_FC and the adjusted *p*-values for two strategies at a given time point (bottom). For *N*-glycan nomenclature, please refer to Supplementary Fig. 5. **C** Galactosylation and **D** glycation index of all feeding strategies at the exponential and stationary time points. Statistical analyses show pairwise comparisons of a given Gal+ strategy with its non-Gal+ counterpart. Adjusted *p*-values were used to assign significance levels, with thresholds defined as *p* < 0.05 (*), *p* < 0.01 (**), and *p* < 0.001 (***). The error bars of the plots represent the standard deviation of the biological replicates.

To dissect the effects of Gal+ and glucose supplementation on *N*-glycan profiles, we first compared clr-transformed glycoform abundances across four feeding strategies: STD and STD+, as well as LoG and LoG+. During the exponential phase (Fig. 5A, Exponential), significant differences (adj. *p*-value < 0.05) were observed between Gal+ supplemented and non-supplemented strategies, indicating that Gal+ strongly influenced *N*-glycan composition. These effects were not diminished by reduced glucose levels, as similar differences were found between LoG+_vs_LoG and STD+_vs_LoG. Notably, comparisons within Gal+ (LoG+_vs_STD+) and non-Gal+ (LoG_vs_STD) strategies revealed no significant differences (statistical comparisons not shown), suggesting that glucose concentration alone had a limited impact on glycosylation at this stage of the bioprocess. In the stationary phase (Fig. 5A, Stationary), comparisons were restricted to samples collected at the same time point (LoG and LoG+ at day 10 due to culture collapse (see Fig. 1A, B), STD and STD+ at day 11). At both time points, Gal+ supplementation led to a significant increase in galactosylated glycoforms. In summary, Gal+ supplementation consistently increased the relative abundance of galactosylated *N*-glycans in both the exponential and stationary phases. This effect was independent of glucose concentration in the medium, indicating that galactosylation is increased by Gal+ availability under the tested strategies.

To evaluate the impact of the HIPDOG-like feeding strategy and Gal+ supplementation on *N*-glycan composition, we compared clr-transformed glycoform abundances between three feeding strategies: HiF, HIP, and HIP+. During the exponential phase (Fig. 5B, Exponential), the HIPDOG-like strategy alone did not result in significant changes in cNISTmAb glycoform abundances when compared to the HiF strategy. However, HIP+ led to a pronounced increase in galactosylated glycoforms, indicating that Gal+ supplementation again is the primary driver of enhanced galactosylation during this growth phase. In the stationary phase (Fig. 5B, Stationary), HIP alone induced a modest decrease in non-galactosylated glycoforms, suggesting some, but limited influence of this feeding strategy on *N*-glycan composition. Notably, the combination of HIPDOG-like feeding with Gal+ (HIP+) produced the highest levels of galactosylated glycoforms observed across all strategies, highlighting its additive effect during the later production phase.

To evaluate the overall effects of the feeding strategies on the levels of galactosylated *N*-glycans and the levels of mAb glycation, we calculated the galactosylation index (Eq. (7)) and the glycation index (Eq. (8)), respectively. As expected, the supplementation of Gal+ increased the total galactosylation of the mAb *N*-glycans in the stationary phase at the final harvest (Fig. 5C, Supplementary Table 8). In the strategies where Gal+ was not supplemented, we observed a decrease in galactosylation during the stationary phase. Interestingly, the decrease in galactosylation was smaller in the low glucose strategies HIP and LoG. The level of mAb glycation was found to differ between feeding strategies during the exponential phase (Supplementary Table 9). We observed that mAb glycation was highest in strategies that received supplemented glucose every day, while in feeding strategies with every-other-day feeding intervals, the glycation was lower (Fig. 5D). At day 5, the viable cell density in HiF, HIP, and HIP+ was lower than in the strategies with every-other-day feeding (Fig. 1A, see Discussion). Because mAb glycation is a non-enzymatic reaction driven by the concentration of free reducing sugars, a lower VCD results in reduced overall glucose uptake, leaving more residual glucose in the medium, thereby increasing the likelihood of mAb glycation. In the stationary phase, the levels of glycated mAb balanced out the differences observed during the exponential phase (Supplementary Table 9). Namely, the feeding strategies with the highest total glycation during the exponential phase (HiF, HIP and HIP+) experienced a decrease in glycated mAb during the stationary phase (Fig. 5D). Due to their more limited glucose replenishments, these strategies therefore also had less glucose available in the medium to allow for more glycation of the mAb (see Fig. 1E, F, Results Carbon source availability determines process characteristics). Finally, increased mAb glycation was observed in the remaining four strategies (STD and STD+, LoG and LoG+). In the LoG and LoG+ strategies, this increase is consistent with prolonged exposure of antibodies present in the medium to glucose during late culture stages, when cell viability and glucose consumption were low, and no substantial dilution by newly secreted mAb occurred (see Figs. 1A, E and 2B, C, Results Carbon source availability determines process characteristics). In conclusion, these results show that the feeding frequency and thus the concentration of available glucose in the medium directly affect mAb glycation levels, with the most pronounced differences occurring during the exponential phase compared to the stationary phase.

## Discussion

In this study, glucose availability had a decisive impact on both process performance and product quality. The STD strategy yielded the highest *q*_P_ and final mAb titer, despite higher lactate levels in comparison to LoG and HIP strategies (Fig. 1). The absence of adverse effects may be attributed to lactate concentrations remaining below 20 mM across all strategies, which are considered favorable in CHO-based processes^31^. While the HIPDOG-like strategy, as described by Gagnon et al.^27^, is typically designed to reduce lactate accumulation without compromising productivity, our results showed that although HIP and HIP+ maintained growth and reduced lactate levels compared to STD, they failed to sustain/increase mAb production. This may be due to insufficient glucose availability in the culture, where the lack of online pH monitoring, especially overnight, hindered timely glucose additions. This caused glucose concentrations to drop below 0.5 g/L, a level considered limiting for CHO cells due to reduced transporter efficiency and increased energetic costs^27,32,33^. We could also confirm that higher glucose availability led to increased glucose consumption, consistent with previous reports in CHO^34^. We believe that these results reflect a cell line-specific preference of NISTCHO for higher glucose concentrations (8 g/L every-other-day). Such preferences have been observed in other CHO cell lines, where elevated glucose feedings maintain productivity while excessive lactate accumulation is prevented by efficient metabolic regulation and substrate utilization^27,31,35^.

Regarding product quality, low-glucose strategies (LoG, HIP) initially showed glycan profiles comparable to STD and HiF during the exponential phase (Fig. 3). However, during the stationary phase, they exhibited increased levels of fucosylated glycoforms (none ⋅ G0F, G0F ⋅ G0F) and a reduction in afucosylated species (G0 ⋅ G0, G0 ⋅ G0F), which may negatively affect ADCC potency^20^. An increase in fucosylated glycoforms under glucose-limited conditions may reflect alterations in nucleotide-sugar metabolism, as GDP-fucose is synthesized from glucose-derived precursors via the GDP-mannose pathway. Previous studies have shown that fucosylation can be sensitive to changes in cellular metabolic state and carbon flux distribution^36,37^. Our results suggest that modulation of nucleotide-sugar availability could contribute to the observed fucosylation patterns and warrants further mechanistic investigation.

Contrary to previous findings^22^, no significant reduction in galactosylation (G1F ⋅ G1F, G1F ⋅ G2F) was observed despite glucose depletion. Instead, we detected a slight increase in the low-abundant G2F ⋅ G2F variant. Given the association between lower fucosylation and enhanced ADCC activity, the shift toward fucosylated species under glucose limitation may be undesirable from a therapeutic efficacy standpoint^20^. Despite differences in glucose feeding, glycation levels of cNISTmAb remained largely unchanged during the stationary phase. The increase in glycation in LoG and LoG+ is particularly unexpected, given that these cultures experienced very low residual glucose concentrations during the later phase in the process (Fig. 1D, E). A plausible explanation lies in the dynamics of the feeding strategy: Bolus glucose additions were applied, creating transient glucose spikes that may have promoted non-enzymatic glycation of the mAb, even though glucose was subsequently depleted. Moreover, during the final 2 days of culture, viable cell density declined sharply, resulting in reduced glucose consumption. Consequently, antibodies already present in the medium were likely exposed to elevated glucose concentrations for an extended period (see Fig. 1A, E), while little to no newly secreted, non-glycated mAb was available to dilute the pool, leading to an overall increase in glycation. Quan et al.^38^, observed a 0.3% increase in glycation per additional g/L of glucose. However, glycation occurs primarily at the heavy chain lysine 98 (HCK98) site in other antibodies^39^, whereas cNISTmAb contains an alanine at this position, which could render it less prone to glycation. In summary, glucose availability strongly influenced process performance, with higher concentrations supporting both growth and productivity, while low-glucose strategies led to earlier process collapse and/or reduced product yields. Despite minimal effects on glycation, lower glucose availability altered *N*-glycosylation profiles in the stationary phase, notably increasing fucosylation.

Our findings indicate that bioprocess performance and product quality under the STD and HiF strategies were comparable in terms of growth as well as glucose and lactate specific rates (Figs. 1 and 2). However, significant differences emerged in terms of productivity: both the average *q*_P_ (Fig. 2B, C) and final cNISTmAb titer (Fig. 1I) were significantly lower under HiF. We hypothesize that the daily feeding led to a transient rise in osmolality, which has been reported to disturb cell homeostasis and productivity, compared to regimens that allow longer adaptation periods between feeds^40^. Additionally, given that these cultures were operated in shaking incubators, daily prolonged handling increased the frequency of temperature drops.

In terms of product quality, the *N*-glycan profiles of STD and HiF clustered closely at both exponential and stationary phases (Fig. 3). Consistent with previous observations^8,41^, we observed decreasing galactosylated glycoforms (G1F ⋅ G1F and G1F ⋅ G2F) over time under these standard feeding strategies.

Despite comparable growth and metabolic rates, the HiF strategy resulted in reduced productivity, likely due to osmolality stress. Moreover, the operational simplicity of every other day feeding reduces handling, while achieving superior end titers with similar CQAs.

The supplementation of Gal+ had no general impact on cell growth or cell-specific rates, in line with previous reports^10,12,13^. However, under nutrient-limited strategies such as LoG+, galactose appeared to serve as an alternative carbon source once glucose and lactate were depleted, supporting extended viability and improved cumulative biomass (Figs. 1 and 2A). This effect was absent in glucose-rich strategies like STD+, where galactose utilization was likely redundant, consistent with previous findings that CHO cells prefer glucose over galactose^9^.

With respect to product quality, Gal+ supplementation led to consistently increased levels of galactosylated glycoforms, as shown by the Galactosylation index (Fig. 5C). These results align with prior reports and manufacturer claims^42,43^, and confirm that Gal+ enhanced *N*-glycan galactosylation regardless of the underlying feeding strategy. Although the exact formulation of Gal+ remains proprietary, we detected both galactose and manganese in significant amounts (Supplementary Table 3), components known to impact galactosylation^8,11,13,14,44^. In LoG+, high galactosylation levels were maintained despite early glucose depletion (Fig. 3), suggesting that galactose alone sustained UDP-galactose synthesis and maintained glycosylation capacity in the Golgi. This is likely due to increased intracellular levels of UDP-galactose via the GALK-GALT pathway and upregulation of galactosyltransferases (*β*-Gal II-IV)^8,12,13,45,46^.

Notably, HIP+ was the only strategy without a time-dependent galactosylation increase (Fig. 4). The absence of a time-dependent galactosylation increase in HIP+ may reflect a saturation or bottleneck in glycan maturation capacity, rather than simply suboptimal energy supply or timing. Indeed, previous studies have shown that increased extracellular galactose (or galactose and Mn^2+^) does not always yield a linear increase in Fc galactosylation—sometimes only a modest shift from G0F to G1F is observed despite elevated intracellular UDP-Gal levels^11,45–47^. This suggests that limitations such as availability or activity of *β*-galactosyltransferases, nucleotide-sugar transport into the Golgi, glycan-processing kinetics, or clone-specific glycosylation capacity may constrain further galactosylation under the conditions of HIP+. Alternatively, HIP+ may impose metabolic or secretory stress, or alter fluxes away from glycosylation, thereby limiting the conversion of G0F to galactosylated forms. Such clone- or process-dependent limitations have been reported before^45–48^.

Gal+ supplementation consistently enhanced antibody galactosylation, which is desirable due to enhanced CDC impact, without negatively impacting cellular performance. Notably, under nutrient-limited strategies, galactose supported extended viability and maintained glycosylation capacity, highlighting its dual role as a carbon source and *N*-glycosylation precursor.

In summary, our study presents a comprehensive investigation of the effects of different bioprocess feeding strategies on the model cell line NISTCHO and on the quality attributes of the produced cNISTmAb. We performed various analyses of the bioprocess parameters and systematically evaluated the differences between *N*-glycan distributions utilizing clr transformation to enable multivariate statistics of compositional data across feeding strategies. Overall, we found that a feeding frequency of every-day versus every-other-day did not affect the monitored CQAs, although higher mAb titers and higher cell-specific productivity were observed with the lowerfrequency feeding. Furthermore, we observed that low levels of glucose had an impact on the non-galactosylated *N*-glycan species, producing a distinct *N*-glycan distribution at the time of the harvest. Finally, we conclude that Gal+ addition increased galactosylation with comparable bioprocess and product quality results, despite the depletion of glucose in two out of three Gal+ addition strategies. These effects were quantitatively reflected in the calculated indices: the overall galactosylation index in the Gal+ strategies was 24%, while the overall glycation index across all feeding strategies at the stationary phase was 5%, providing a clear summary of how feeding strategies influenced CQAs. These insights contribute to a deeper understanding of how targeted modifications of feeding strategies can be used to fine-tune monoclonal antibody glycosylation profiles and improve bioprocess outcome in CHO cell-based production systems.

## Methods

### Cell culture

NISTCHO (RGTM 10197) is a recombinant CHO cell line (NIST, Gaithersburg, MD, USA; MilliporeSigma, MA, USA), which expresses a non-originator NISTmAb product, cNISTmAb (for nomenclature see Supplementary Table 1 as proposed by Cleveland et al.^49^). Cells were thawed and passaged in EX-CELL CD CHO Fusion medium (Merck, Darmstadt, Germany) every 3–4 days and cultivated in a humidified incubator (Kuhner, Birsfelden, Switzerland) at 37.0 °C, 220 rpm, 7.0% CO_2_, 85.0% relative humidity, with an orbital shaking diameter of 25.0 mm. Before the start of the fed-batch culture, the medium was switched to EX-CELL Advanced CHO Fed-batch medium (Merck). Fed-batch cultures were inoculated after three cell passages and cultivated with an initial working volume of 20.0 mL using a seeding concentration of 0.4 × 10^6^ cells/mL in 50 mL TubeSpin bioreactors (Techno Plastic Products, Trasadingen, Switzerland). A feed mixture was used, consisting of 66.0% EX-CELL Advanced CHO Feed 1 (Merck) and 33.0% Cellvento 4Feed COMP (Merck), as recommended by the manufacturer (MilliporeSigma), who co-developed the cell line. The amino acid composition of the basal and feed media was analysed in-house, and concentrations are provided (see Supplementary Table 2). Glucose levels were replenished to the target concentration using 45.0% D-Glucose solution (Sigma-Aldrich, MO, USA), that was diluted to a of 25.0% solution. For co-supplementation, the protein quality supplement EX-CELL Glycosylation Adjust, Gal+ (Merck) was used. Since galactose and manganese (Mn^2+^) were suspected to be the key contributors to product quality modulation, their concentrations in the Gal+ supplement were quantified in-house and are reported (see Supplementary Table 3). The cultures were terminated when viability dropped below 60% or after 11 days.

### Fed-batch feeding strategies

The feeding strategies implemented in this study involved variations in glucose concentrations, feed intervals, and volumes, and optional Gal+ supplementation, as detailed in Table 1. Feeding events for the main feed mixture and Gal+ commenced day 3 post-inoculation for all strategies, independent of the glucose regimen. To avoid introducing osmolality-related confounders due to different feed volumes, all volumetric additions were normalized across feeding strategies. While the glucose addition volume varied depending on the target concentration (replenishment to 2.0, 4.0, or 8.0 g/L), the total volume added per feeding event was kept constant by adjusting with nuclease-free water. The protocol for the fed-batch feeding strategies, including detailed feeding schedules, target glucose concentrations, and volumetric adjustments for all strategies, is provided in the accompanying Excel file deposited on Zenodo. The standard method (STD) involved a 48-h feed interval, using 5.0% (v/v) of the feed mixture where glucose was replenished to 8.0 g/L. The second strategy was based on the STD strategy, with the addition of 0.2% (v/v) Gal+ supplement every other day. This corresponds to final concentrations of an additional 1.25 mM galactose and 0.29 μM Mn^2+^ in the culture per feed. For the low glucose (LoG) strategy, the target glucose concentration was reduced to 4.0 g/L, halving the glucose replenishment compared to STD. The LoG+ strategy combined this reduced glucose replenishment with the same Gal+ supplementation as mentioned above. The higher frequency feed (HiF) strategy evaluated feeding daily, with a reduced feeding volume of 2.5% (v/v) and glucose replenished to 4.0 g/L. The HIP strategy explored a HIPDOG (HI-end pH-controlled Delivery Of Glucose)-like feeding approach, as established by Gagnon et al.^27^. To approximate the HIPDOG strategy in uncontrolled pH settings, such as the TubeSpin bioreactors, glucose was added only when culture pH reached or exceeded 7.2. This pH threshold was used as a proxy for glucose depletion. At each feeding event, glucose was supplemented to a final concentration of 2.0 g/L. This manual, pH-guided approach aimed to limit glucose availability and reduce lactate accumulation. The feed mixture volume was replenished to 2.5% (v/v) every 24 h. The HIP+ strategy combined this pH-triggered glucose feeding approach with additional Gal+ supplementation, as detailed above. The experiments were conducted across two sequential fed-batches: Strategies STD, STD+, LoG, and LoG+ were each performed with four biological replicates, while HiF, HIP, and HIP+ included three biological replicates (as outlined in Table 1).

### In-process cell culture measurements

Fed-batch cultures were sampled daily prior to feeding, and consistent sampling and feeding volumes were maintained across all strategies throughout the entire process. Viable cell density (VCD) and viability were measured using an automated cell counter (ViCell XR, Beckman Coulter, CA, USA). Glucose concentrations were quantified using a glucose meter (Contour Next, Ascensia Diabetes Care, Basel, Switzerland) according to the manufacturer’s instructions and corrected with a standard curve. The pH was monitored once daily by using a micro pH electrode (Mettler Toledo, OH, USA). The IgG concentration in the supernatant was determined by Bio-Layer Interferometry on an Octet RED96e (Sartorius, Göttingen, Germany) using Protein A biosensors (Sartorius). The cNISTmAb concentrations were calculated using a standard curve prepared with NISTmAb (RM8671, Merck), ranging from 1.5 to 100.0 μg/mL. Extracellular lactate levels were measured using a colorimetric lactate assay kit (MAK064, Sigma-Aldrich) every 48 h starting at day 3 according to the manufacturer’s protocol. Sampling of 1 mL supernatant for product quality analysis was carried out during the exponential phase for all strategies at day 5 and during the stationary phase at day 10 (strategies LoG and LoG+) or 11 (strategies STD, STD+, HiF, HIP, HIP+).

### Data evaluation of cell-specific rates

To determine cell-specific rates of growth, metabolite consumption/production, as well as product formation, the following equations were applied. The specific growth rate *μ* was calculated over time by

1 $$\mu =\frac{\Delta \,\mathrm{ln}\,(\,\mathrm{VCD})}{\Delta t}$$

where ΔVCD is the change in the natural logarithm of viable cell density (10^6^ cells ⋅ mL^−1^) between two consecutive time points, and Δt is the corresponding time interval in days (d).

The integrated viable cell density (IVCD) was calculated as the trapezoidal area under the VCD curve between two time points by Eq. (2).

2 $${{\rm{IVCD}}}_{i}=\frac{1}{2}\left({{\rm{VCD}}}_{i-1}+{{\rm{VCD}}}_{i}\right)\cdot ({t}_{i}-{t}_{i-1})$$

IVCD_i_ is the incremental IVCD calculated for interval *i* expressed as 10^6^ cells ⋅ d ⋅ mL^−1^. VCD_i-1_ and VCD_i_ were taken at the previous and current time points, respectively, and t is the time interval (d).

Cumulative IVCD up to timepoint *i* was given by Eq. (3).

3 $${{\rm{IVCD}}}_{{\rm{sum}},i}=\mathop{\sum }_{j=2}^{i}{{\rm{IVCD}}}_{j}$$

where IVCD_j_ represents the IVCD between time points *j* − 1 and *j*.

The cell-specific rate for lactate (${q}_{{{\rm{LAC}}}_{i}}$) was calculated with Eq. (4) for each consecutive time point in 48 h intervals.

4 $${q}_{{{\rm{LAC}}}_{i}}=\frac{\Delta {{\rm{LAC}}}_{i}}{\Delta {{\rm{IVCD}}}_{i}}$$

Where ${q}_{{{\rm{LAC}}}_{i}}$ is the change in extracellular lactate concentration, pmol ⋅ cell^−1^ ⋅ d^−1^.

Similarly, the cNISTmAb productivity per cell can be described as in Eq. (5) for each consecutive time point in 24 h intervals.

5 $${q}_{{{\rm{P}}}_{i}}=\frac{\Delta {{\rm{P}}}_{i}}{\Delta {{\rm{IVCD}}}_{i}}$$

Where ${q}_{{{\rm{P}}}_{i}}$ is the productivity given as pg ⋅ cell^−1^ ⋅ d^−1^.

Lastly, the specific glucose consumption rate (${q}_{{{\rm{GLC}}}_{i}}$) was calculated using a linear regression (Eq. (6)). The ${q}_{{{\rm{GLC}}}_{i}}$ was determined separately for each strategy based on its respective feeding interval.

6 $${c}_{{{\rm{GLC}}}_{i}}={q}_{{{\rm{GLC}}}_{i}}\cdot {{\rm{IVCD}}}_{{\rm{sum}},i}+d+{\varepsilon }_{i}$$

The ${c}_{{{\rm{GLC}}}_{i}}$ represents the measured extracellular glucose concentration at the timepoint *i*. *d* is the intercept of the linear regression, and *ϵ*_i_ is the residual error between observed and fitted glucose concentrations. The slope of the model corresponds to ${q}_{{{\rm{GLC}}}_{i}}$, assuming a constant glucose consumption rate within the interval.

### Intact protein analysis

Intact protein analysis was performed according to the previously published dilute-and-shoot methodology developed by Regl et al.^50^. For IP-RP-HPLC-MS, aliquots of the cell culture supernatants containing cNISTmAb were thawed and centrifuged for 3 min at 4 °C with 14,000 rcf. Then, 30,000 ng of cNISTmAb were buffer exchanged to 150 mM AmAc (Fisher Scientific, Loughborough, UK) using Amicon 10 kDa mass cutoff filters (Merck Millipore, Tullagreen, Cork, Ireland). Afterwards, the obtained volume was readjusted to 200 μL (cNISTmAb target concentration of 150 ng/μL). After transferring 90 μL of the sample to an HPLC vial and adding 10 μL ribonuclease A (1 mg mL^−1^, Fisher Scientific), the sample was thoroughly mixed and placed in the HPLC auto-sampler for analysis. For the PNGase F digests, 1 μL of PNGase F (500 units, NEB P0705S, PNGase F Glycerol-free, New England Laboratories, Frankfurt am Main, Germany) was added to the remaining volume of each sample. After incubation for 3 h at 37 °C under gentle shaking at 750 rpm, 90 μL were transferred to HPLC vials and 10 μL of ribonuclease A added before analysis. Ultrapure water for the experiments was produced in-house, using a MilliQ Integral 3 water purification system by Merck/Millipore (Billerica, MA, U.S.A.) To ensure reproducibility, HPLC-MS measurements for each sample were performed in technical triplicate. Furthermore, the NISTmAb reference material (RM8671; 135 ng/μL in 150 mM AmAc) was injected as a quality control (QC) standard every 30 injections. Recorded chromatographic and MS data, including retention times, mass accuracies, and fractional *N*-glycoforms abundances, remained stable in the QC samples throughout the measurement sequence. The relative standard deviation (RSD) values for mass accuracies and fractional *N*-glycovariant abundances of the two most abundant *N*-glycovariants in the QC were comparable to previously reported data^41^ (see Supplementary Fig. 8, Supplementary Table 7). As good repeatability was already demonstrated by Boettinger et al. for this method, the comparable RSDs observed here confirm the repeatability of our HPLC-MS measurements. Analysis of the non-digested and PNGase F digested cNISTmAb and NISTmAb samples was carried out on a Dionex Ultimate 3000 UHPLC (Thermo Fisher Scientific, Germering, Germany) directly coupled to a Q Exactive^TM^ Plus Hybrid Quadrupole Orbitrap^TM^ mass spectrometer (both Thermo Fisher Scientific, Bremen, Germany). The chromatographic separation was achieved by using a MAbPac RP 5 μm, 2.1 × 50 mm column (Thermo Fisher Scientific, Sunnyvale, CA, USA) with mobile phase A containing water with 0.050% trifluoroacetic acid (TFA, Fisher Scientific, Loughborough, UK) and mobile phase B containing acetonitrile (Fisher Scientific) with 0.050% TFA. A gradient-based elution was performed for the separation: After holding mobile phase B at 25.0% B for the first minute, B was increased to 35.0% B in 0.50 min. Afterwards, B was further raised to 41.7% in 2.5 min. After a washing step with 99% eluent B for 2.0 min at a flow rate of 600 μL min^−1^, B was held at a flow rate of 600 μL min^−1^ for 3.0 min at 25% B. Then, the flow rate was decreased back to 300 μL min^−1^ at 25% B for 1.0 min to re-equilibrate the column for the next injection. The starting flow rate was set to 300 μL min^−1^, the column temperature to 70 °C, UV data were acquired at 214 nm wavelength, and 5 μL of sample was injected per run. The parameters for the HESI II ion source (Thermo Fisher Scientific, Bremen, Germany) can be found in Table 2. To confirm that the changes in HESI source parameters did not affect MS performance, previously analyzed samples and the QC standards were re-injected. The obtained mass spectra showed highly consistent results across all compared runs. The MS settings can be found in Table 3.

**Table 2:** HESI source parameters

Parameter	Setting
Spray voltage	4.000 or 4.500 kV
Sheath gas flow rate	15 or 50 arbitrary units (au)
Aux gas flow rate	5 or 10 au
Sweep gas flow rate	0 au
Capillary temperature	300 °C
S-lens RF level	80.0 or 100.0
Aux gas heater temperature	250 °C

**Table 3:** Mass spectrometer parameters

Parameter	Setting
Scan type	Full MS
Polarity	Positive
Resolution setting	17,500 @ *m*/*z* 200
Scan range	1800.0–5500.0 m/z
Microscans	10
AGC target	3 × 10^6^
Maximum injection time	150 ms
In-source CID	80.0 eV

### Analysis of *N*-glycosylation and mAb glycation

The peak of the intact mAb used for *N*-glycan analysis within each sample (Supplementary Fig. 4) was selected based on the visual inspection of the raw mass spectrum files using Thermo Scientific Excalibur—Qual Browser (v4.2.28.14) (Thermo Fisher Scientific) by defining a retention time window.

Prior to quantification, the combinations of *N*-glycans attached to the mAb present in each sample were checked by deconvolution of the raw mass data in the Thermo Scientific^TM^ BioPharma Finder^TM^ software version 3.0 (Thermo Fisher Scientific, San Jose, CA, USA) using its ReSpect algorithm and comparison with calculated theoretical mass values (Supplementary Figs. 6 and 7). For the *N*-glycan annotation, we use the symbol “⋅” to denote the two *N*-glycans of an intact antibody: for example, “G1F ⋅ G0F” represents cNISTmAb with two bi-antennary glycans carrying one galactosylated (G1F) and one non-galactosylated (G0F) species. The annotation using “⋅” was used previously for the same purposes^26^.

After the *N*-glycan peaks were annotated, the raw mass spectrum files were converted to .mzml format using ThermoRawFileParser (v1.7.3). Quantification of *N*-linked glycosylation and of glycation from mass spectrometry data was conducted in R (v4.3.0) with the R-package fragquaxi (v1.0) (https://github.com/cdl-biosimilars/fragquaxi)^41,51^. After the quantification of the *N*-glycan abundances by extracted ion current integration, the raw abundances were converted to fractional abundances (all glycoforms for a single technical replicate sum up to 100%). Mass spectra of PNGase F-digested samples were used to quantify the abundance of glucose attached to the mAb. While mAb glycation was also quantified as a product quality (Fig. 5D), this data was also used for the correction of the hexosylation bias in the *N*-glycan data, using the Python-based library CAFOG (v 1.0) (https://github.com/cdl-biosimilars/cafog)^26^.

The galactosylation index was calculated to quantify the degree of galactose occupancy on *N*-glycans across galactosylation sites^52^. For each glycoform *j*, the number of galactose residues *g*_*j*_ and the number of possible galactosylation sites *d*_*j*_ were determined by parsing the glycan composition. The contribution of each glycoform was weighted by its fractional abundance *A*_*j*_. The galactosylation index was calculated for each sample as:

7 $${\rm{Galactosylation\; index}}=\left(\frac{{\sum }_{j=1}^{n}{A}_{j}\cdot {g}_{j}}{{\sum }_{j=1}^{n}{A}_{j}\cdot {d}_{j}}\right)\times 100$$

where: *A*_*j*_ is the corrected fractional abundance of glycoform *j*, *g*_*j*_ is the number of galactose residues on glycoform *j*, *d*_*j*_ is the number of possible galactosylation sites on glycoform *j*, *n* is the number of glycoforms observed in the sample. Each glycoform was considered as a pair of *N*-glycans, with a default of two galactosylation sites per glycan.

The glycation index quantifies the extent of hexose attachment (glycation) to the antibody, reflecting the average occupancy of glycation sites across all glycoforms in a sample. The glycation index was calculated for each sample as:

8 $${\rm{Glycation\; index}}=\left(\frac{{\sum }_{i=1}^{n}{g}_{i}\cdot {f}_{i}}{{\sum }_{i=1}^{n}{s}_{i}\cdot {f}_{i}}\right)\times 100$$

where: *g*_*i*_ is the number of hexose units attached to glycoform *i* (0, 1, 2, or 3), *f*_*i*_ is the fractional abundance of glycoform *i* in the sample (with ∑*f*_*i*_ = 100), *s*_*i*_ is the maximum possible glycation sites for glycoform *i* (fixed at 3 for all glycoforms), *n* is the total number of glycoforms detected in the sample.

### Statistical analyses

All statistical analyses were performed in R (v4.4.3). For each response variable, normality of residuals was assessed using the Shapiro-Wilk test (R package stats, v4.4.3), and homogeneity of variances was evaluated using Levene’s test (R package car, v3.1.3). When assumptions of normality and equal variances were satisfied, a one-way ANOVA (also from stats) was applied to test for differences between feeding strategies for each growth phase, followed by Tukey’s Honestly Significant Difference (HSD) post hoc test (also from stats). Normality was violated only for the glycation comparison within one feeding strategy; therefore, this specific case was analyzed using the non-parametric Kruskal–Wallis test, followed by Dunn’s post hoc test with multiple-comparison correction (both from R package rstatix, v0.7.2). Adjusted *p*-values from post hoc comparisons were used to determine significance, with thresholds defined as *p* < 0.05 (*), *p* < 0.01 (**), and *p* < 0.001 (***).

To analyze fractional abundances of *N*-glycans, respective data were transformed using centered log-ratio (clr() function (R package compositions, v2.0-9)), since glycan abundances are expressed as fractional abundances that sum to a constant (compositional data)^28–30^. This transformation projects data from the constrained simplex space into an unconstrained Euclidean space by computing, for each component, the logarithm of its ratio to the geometric mean of all components, thereby enabling the use of standard linear models, principal component analysis, and correlation analyses. A common limitation of CLR is its inability to handle zero components, which typically require small-value replacement prior to log transformation. In our dataset, all quantified glycoforms were detected across samples, and zero abundances were not present; therefore, no imputation or replacement was necessary. Due to the fact that the relationship to the original data unit is lost after clr transformation, fractional abundance is shown without the clr transformation in the main figures, while clr-transformed data is shown in Supplementary Figs. 9, 10 and 11. PCA was performed (on clr-transformed *N*-glycan data), using the prcomp() function from R stats (v4.3.0), to identify major patterns of variation across samples. Spearman correlations between samples were computed on clr-transformed *N*-glycan data using cor(..., method = “spearman”, use = “pairwise.complete.obs”) using stats. The input matrix was organized with glycoforms as rows and samples as columns (as is common for omics data^53^), such that each correlation coefficient reflects the similarity of glycan profiles between two samples. Multivariate differences were assessed using PERMANOVA (R package vegan, v2.7-2) with Euclidean distances and 999 permutations to test for effects of culture phase and feeding strategy (dist.eu ~ phase + strategy), and their interaction (dist.eu ~ phase * strategy). Multivariate homogeneity of group dispersion was evaluated using betadisper to confirm that observed differences were not driven by unequal within-group variance.

To statistically compare strategies and sampling time points, linear models (R package limma, v3.58.1^54^) were fitted on clr-transformed *N*-glycan abundances as responses and experimental feeding strategy and time point as predictors. Contrasts were specified to test differences between (i) time points within the same feeding strategy (time effects, Results Fucosylation increases in the stationary phase independent of feeding strategy) and (ii) feeding strategies at the same time point (feeding strategy effects, Results Availability of glucose and galactose determines antibody glycation and galactosylation). Plots were generated in R and assembled into multi-panel figures using Inkscape (https://inkscape.org/). Full analysis code for reproducibility is available on GitHub (Code availability).

## Supplementary information

Supplementary information

## Data Availability

All raw data generated during the bioprocess analyses, including raw mass spectrometry files and a detailed fed-batch protocol (for all feeding strategies), have been deposited in Zenodo and are publicly available at 10.5281/zenodo.17046013.

## Code availability

Full analysis code for analysis reproducibility is available at https://github.com/csbg/DGTX_feeding_strategies_nistcho.
