# Supplementary material for: Effects of feeding strategies on culture performance and product quality in NISTCHO
Source: NPJ Syst Biol Appl. 2026 Mar 19;12:67. doi: 10.1038/s41540-026-00686-3 (PMC13168316; doi:10.1038/s41540-026-00686-3)
Supplement: Supplementary file 1 — Supplementary information [file 41540_2026_686_MOESM1_ESM.pdf]

Supplementary Information

NISTCHO nomenclature

Supplementary Table 1. NIST recommended nomenclature convention for the NISTCHO cell line and its product monoclonal antibody, cNISTmAb [27].

Prefix	University	XXXX Identifier	Lab	YY Identifier	ZZ Identifier	Naming convention
NISTCHO	BOKU University	BOKU	Nicole Borth	NB	W2	NISTCHO-BOKU-NB-W2
cNISTmAb	BOKU University	BOKU	Nicole Borth	NB	W2	cNISTmAb-BOKU-NB-W2

Amino acid composition of medias

Supplementary Table 2. Amino acid composition of the used medias. The feed mixture consisted of 66% EX-CELL Advanced CHO Feed 1 (Merck) and 33% Cellvento 4Feed COMP (Merck). Measured in-house by HPLC.

Amino acid	Advanced Basal [mM]	Advanced Feed 1 [mM]	Cellvento 4Feed [mM]	Feed mix [mM]
Asp	8.88	33.13	20.66	28.02
Glu	1.47	7.92	197.29	72.19
Asn	4.74	11.67	26.89	17.04
Ser	5.12	26.88	92.39	49.90
Gln	–	0.00	0.00	0.00
His	1.17	3.49	9.26	5.20
Gly	0.91	0.53	0.20	0.49
Thr	3.04	9.53	31.20	17.22
Arg	5.03	6.61	21.29	11.94
Ala	0.72	0.44	0.14	0.44
Tyr	1.09	2.76	0.70	2.04
Val	3.01	14.09	63.18	31.56
Met	1.01	3.86	6.31	4.55
Trp	0.92	3.06	8.54	4.15
Phe	1.38	6.81	26.24	13.32
Ile	2.63	14.32	69.77	34.09
Leu	4.08	25.68	59.76	38.71
Lys	3.05	8.87	32.39	17.02
Pro	2.65	8.69	28.71	15.65

GAL+ supplement analysis

Supplementary Table 3. EX-CELL® Glycosylation Adjust (GAL$^+$) composition (undiluted).

Component	Concentration [mM]	Detection method
Galactose	626.30	In-house GC–MS
Manganese	0.145	In-house MP–AES

IVCD all pairwise statistics

Supplementary Table 4. Pairwise comparisons between IVCD results of the feeding strategies. Reported are mean differences (diff), lower and upper 95% confidence intervals (lwr, upr), adjusted p-values (p adj), and significance levels (p <0.05 (*), p <0.01 (**), and p <0.001 (***)).

Group 1	Group 2	Diff	Lwr	Upr	p adj	Significance
STD+	STD	-21.42	-200.90	158.06	1.00E+00	ns
HiF	STD	-59.50	-253.35	134.36	9.44E-01	ns
HIP+	STD	-78.54	-272.40	115.31	8.25E-01	ns
HiF	STD+	-38.08	-231.93	155.78	9.94E-01	ns
HIP+	STD+	-57.12	-250.98	136.73	9.54E-01	ns
HIP	LoG+	-67.00	-260.86	126.86	9.06E-01	ns
HIP+	HiF	-19.05	-226.29	188.20	1.00E+00	ns
HiF	LoG+	288.71	94.86	482.57	1.75E-03	**
HIP+	LoG+	269.67	75.81	463.53	3.47E-03	**
LoG	STD	-891.09	-1070.56	-711.61	5.32E-11	***
LoG+	STD	-348.21	-527.69	-168.73	8.47E-05	***
HIP	STD	-415.21	-609.07	-221.36	2.36E-05	***
LoG	STD+	-869.66	-1049.14	-690.19	8.15E-11	***
LoG+	STD+	-326.79	-506.27	-147.31	1.85E-04	***
HIP	STD+	-393.79	-587.65	-199.93	4.72E-05	***
LoG+	LoG	542.87	363.40	722.35	1.65E-07	***
HiF	LoG	831.59	637.73	1025.45	6.31E-10	***
HIP	LoG	475.87	282.01	669.73	3.61E-06	***
HIP+	LoG	812.54	618.68	1006.40	9.22E-10	***
HIP	HiF	-355.72	-562.96	-148.47	3.71E-04	***
HIP+	HIP	336.67	129.43	543.91	6.93E-04	***

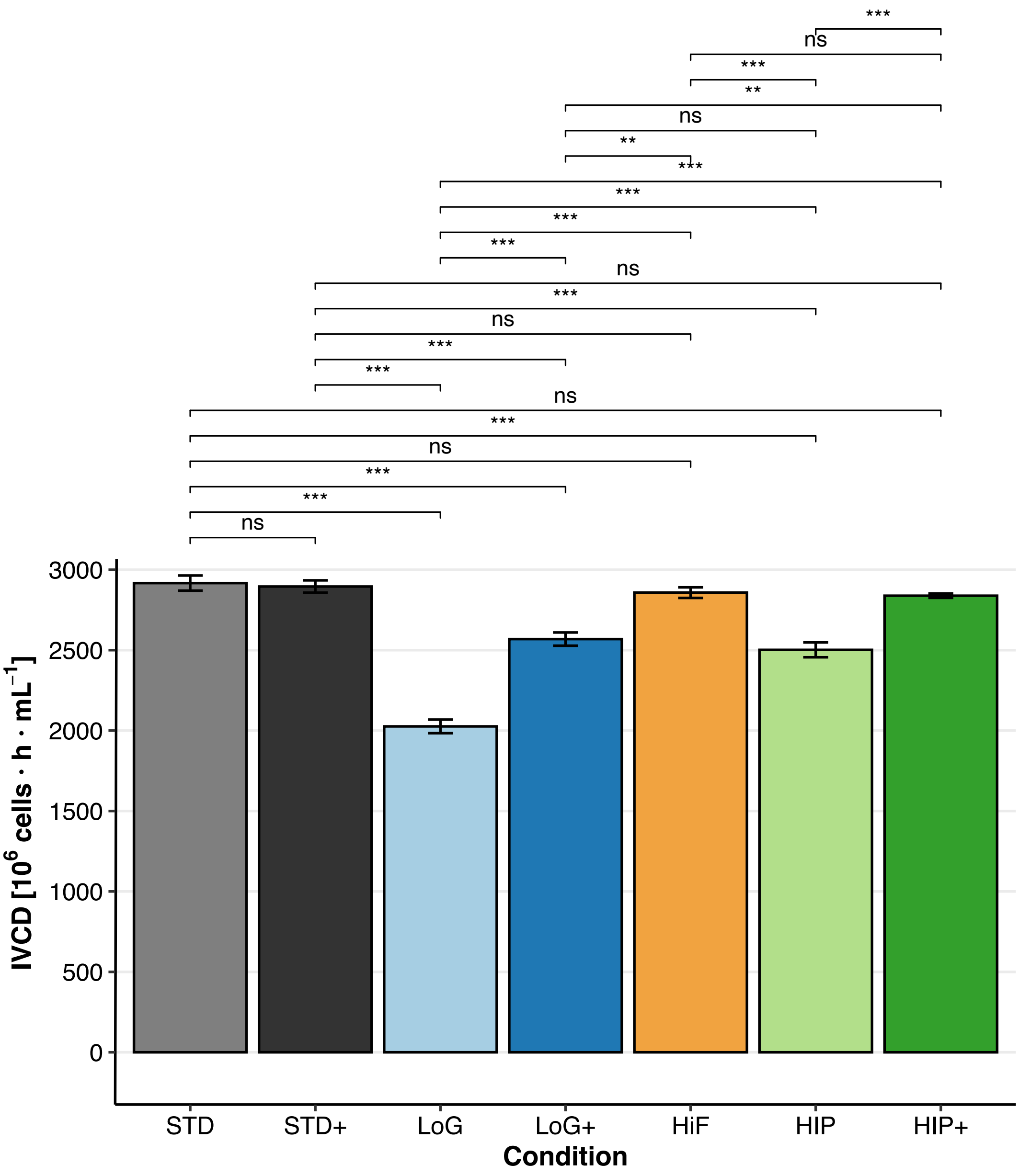

Supplementary Figure 1. All pairwise observations for the IVCD. Adjusted p-values were used to assign significance levels, with thresholds defined as p <0.05 (*), p <0.01 (**), and p <0.001 (***). Error bars represent the standard error of the biological replicates.

Final titers all pairwise statistics

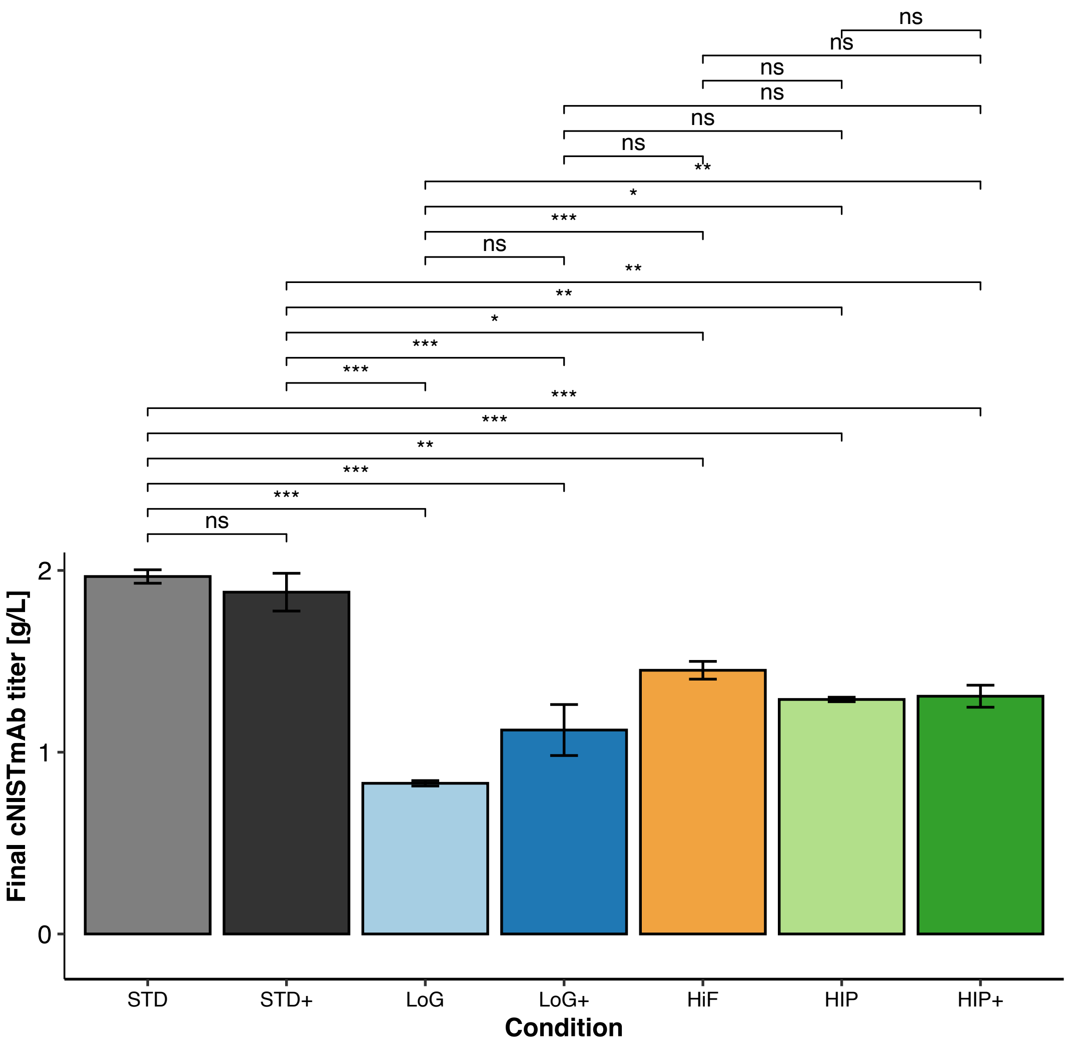

Supplementary Figure 2. Final titers with all pairwise observations. Adjusted p-values were used to assign significance levels, with thresholds defined as p <0.05 (*), p <0.01 (**), and p <0.001 (***). Error bars represent the standard error of the biological replicates.

Supplementary Table 5. Pairwise comparisons between feeding strategies for final cNISTmAb titers. Reported are mean differences (diff), lower and upper 95% confidence intervals (lwr, upr), adjusted p-values (p adj), and significance levels ($p < 0.05$ (*), $p < 0.01$ (**), and $p < 0.001$ (***)).

Group 1	Group 2	Diff	Lwr	Upr	p adj	Significance
STD+	STD	-86.18	-443.90	271.54	9.82E-01	ns
LoG+	LoG	293.00	-64.72	650.72	1.53E-01	ns
HiF	LoG+	328.77	-57.61	715.15	1.27E-01	ns
HIP	LoG+	168.21	-218.17	554.59	7.75E-01	ns
HIP+	LoG+	186.01	-200.37	572.39	6.89E-01	ns
HIP	HiF	-160.57	-573.63	252.49	8.50E-01	ns
HIP+	HiF	-142.77	-555.83	270.29	9.06E-01	ns
HIP+	HIP	17.80	-395.26	430.86	1.00E+00	ns
HiF	STD+	-429.70	-816.08	-43.32	2.37E-02	*
HIP	LoG	461.21	74.83	847.59	1.36E-02	*
HiF	STD	-515.87	-902.26	-129.49	5.11E-03	**
HIP	STD+	-590.27	-976.65	-203.88	1.35E-03	**
HIP+	STD+	-572.47	-958.85	-186.08	1.85E-03	**
HIP+	LoG	479.01	92.63	865.39	9.91E-03	**
LoG	STD	-1137.65	-1495.37	-779.93	7.63E-08	***
LoG+	STD	-844.65	-1202.37	-486.93	6.24E-06	***
HIP	STD	-676.44	-1062.82	-290.06	2.95E-04	***
HIP+	STD	-658.64	-1045.02	-272.26	4.02E-04	***
LoG	STD+	-1051.47	-1409.19	-693.75	2.56E-07	***
LoG+	STD+	-758.47	-1116.19	-400.75	2.69E-05	***
HiF	LoG	621.77	235.39	1008.15	7.69E-04	***

Average q_P all pairwise statistics

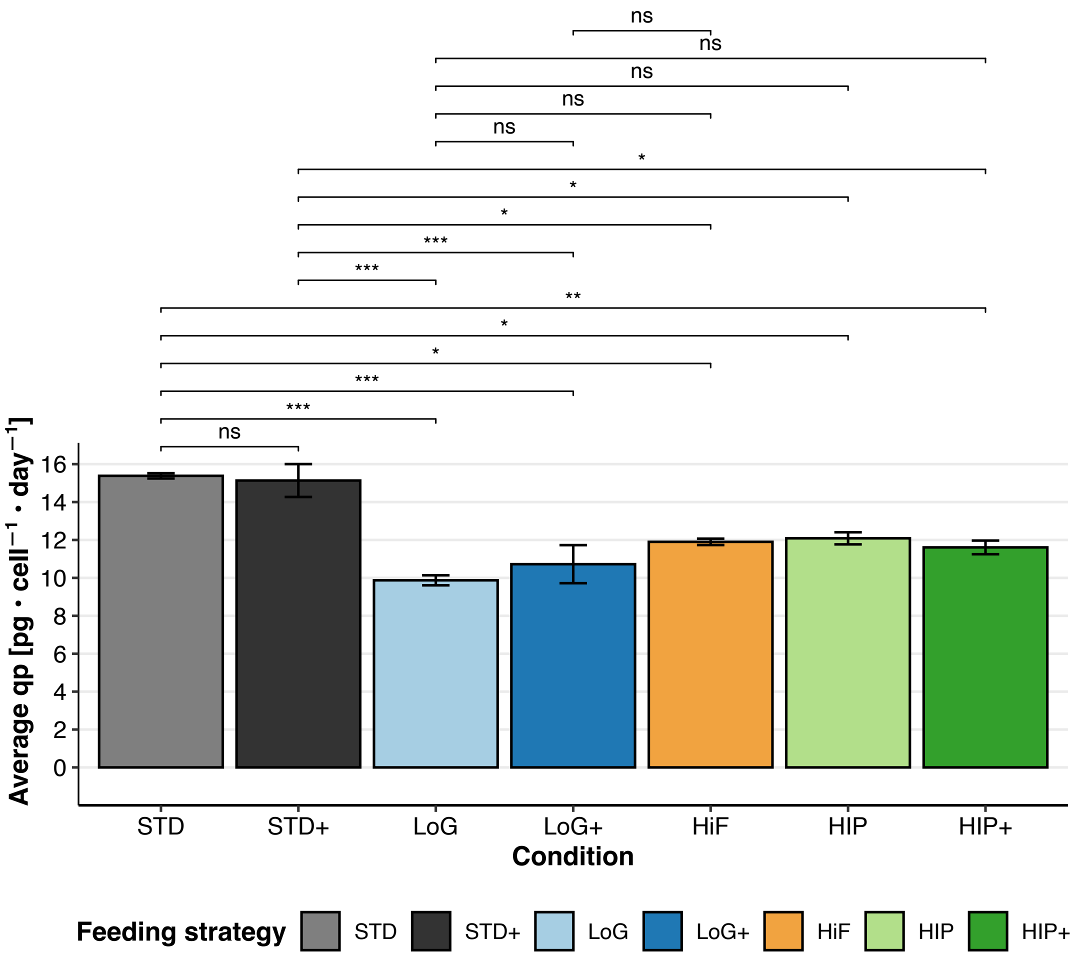

Supplementary Figure 3. Average qp with all pairwise observations. Adjusted p-values were used to assign significance levels, with thresholds defined as p <0.05 (*), p <0.01 (**), and p <0.001 (***). Error bars represent the standard error of the biological replicates.

Supplementary Table 6. Pairwise comparisons between feeding strategies for average cell-specific productivity (q_p). Reported are mean differences (Diff), lower and upper 95% confidence intervals (lwr, upr), adjusted p-values (p adj), and significance levels (p <0.05 (*), p <0.01 (**), and p <0.001 (***)).

Group 1	Group 2	Diff	Lwr	Upr	p adj	Significance
STD+	STD	-0.01	-0.12	0.10	1.00E+00	ns
LoG+	LoG	0.04	-0.08	0.15	9.35E-01	ns
HiF	LoG	0.08	-0.04	0.21	2.90E-01	ns
HIP	LoG	0.09	-0.03	0.21	2.08E-01	ns
HIP+	LoG	0.07	-0.05	0.19	4.59E-01	ns
HiF	LoG+	0.05	-0.07	0.17	8.24E-01	ns
HIP	LoG+	0.06	-0.06	0.18	7.11E-01	ns
HIP+	LoG+	0.04	-0.08	0.16	9.46E-01	ns
HIP	HiF	0.01	-0.12	0.14	1.00E+00	ns
HIP+	HiF	-0.01	-0.14	0.12	1.00E+00	ns
HIP+	HIP	-0.02	-0.15	0.11	9.98E-01	ns
HiF	STD	-0.15	-0.27	-0.02	1.30E-02	*
HIP	STD	-0.14	-0.26	-0.02	2.01E-02	*
HiF	STD+	-0.13	-0.26	-0.01	2.33E-02	*
HIP	STD+	-0.13	-0.25	-0.01	3.57E-02	*
HIP+	STD+	-0.15	-0.27	-0.03	1.17E-02	*
HIP+	STD	-0.16	-0.28	-0.04	6.45E-03	**
LoG	STD	-0.23	-0.34	-0.12	4.12E-05	***
LoG+	STD	-0.19	-0.31	-0.08	3.27E-04	***
LoG	STD+	-0.22	-0.33	-0.11	7.42E-05	***
LoG+	STD+	-0.18	-0.30	-0.07	6.11E-04	***

UV-VIS Chromatograms of all cNISTmAb samples

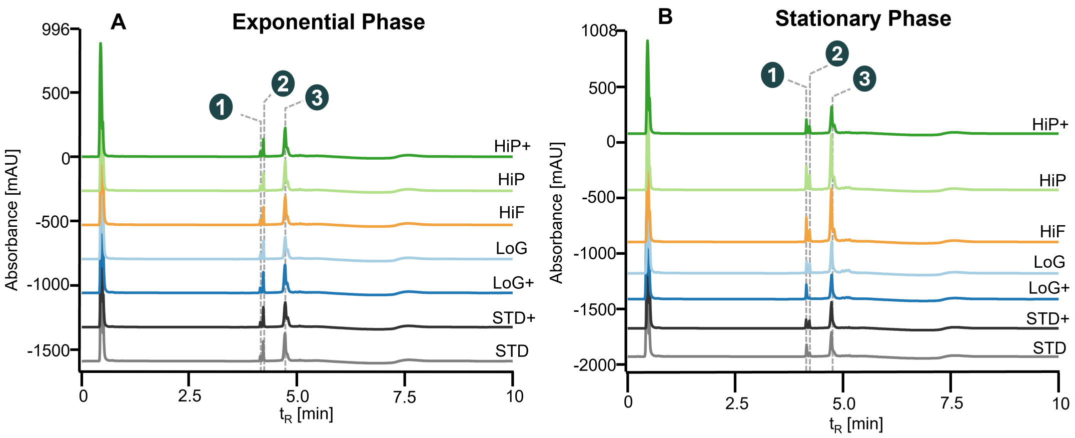

Supplementary Figure 4. IP-RP-HPLC-UV-VIS Chromatograms of all cNISTmAb bioprocessing samples from the different feeding strategies, with peak 1 representing the free light chain of the mAb, peak 2 containing the light chain dimer and peak 3 consisting of the fully formed antibody in every chromatogram for the exponential phase (A) and for the stationary phase (B).

Types of *N*-glycans

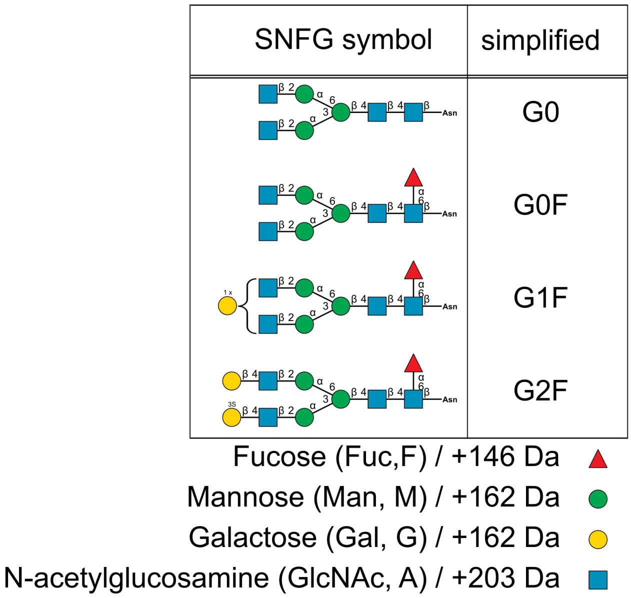

Supplementary Figure 5. Types of *N*-glycans and their nomenclature according to Symbol Nomenclature for Glycans (SNFG). *N*-glycans added to the antibody at Asn-X-Ser or Thr sequeons are of complex type and contain the common core Man3GlcNAc2Asn.

Deconvoluted mass spectrum of the STD sample in the exponential phase

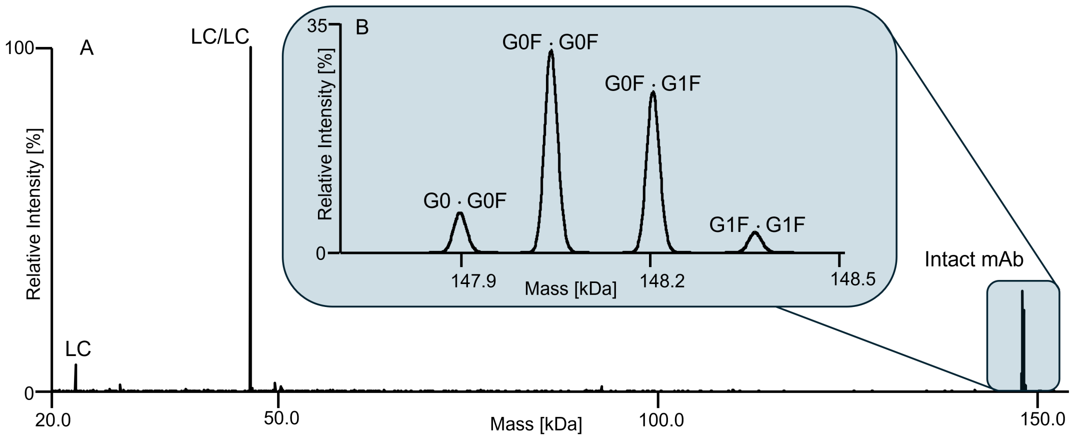

Supplementary Figure 6. Deconvoluted zero-charge mass spectrum of the STD sample in the exponential phase, using the ReSpect algorithm in the BioPharma Finder 3.0 software by Thermo Fisher Scientific. A displays the deconvoluted masses of the free light chain (LC), the light chain dimer (LC/LC) and the fully formed cNISTmAb antibody, B provides a zoom-in on the masses of the main *N*-glycovariants present on the intact antibody.

Deconvoluted mass spectrum of the STD sample in the stationary phase

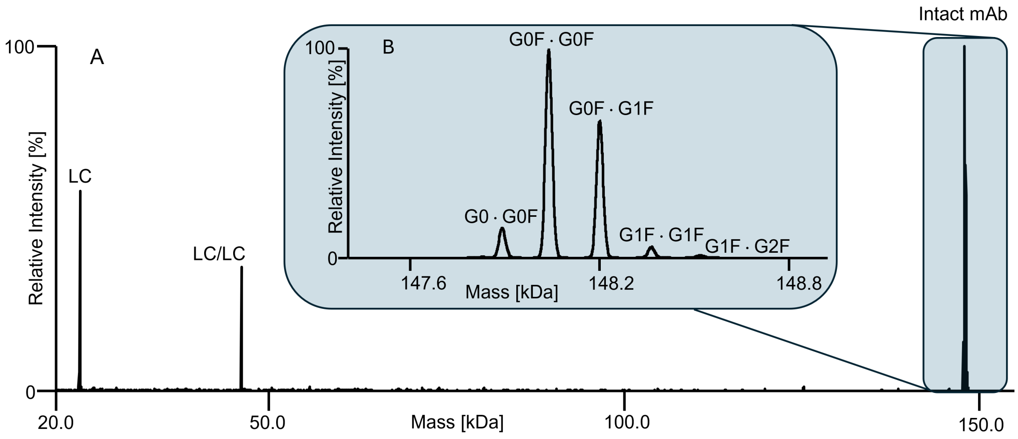

Supplementary Figure 7. Deconvoluted zero-charge mass spectrum of the STD sample in the stationary phase, using the ReSpect algorithm in the BioPharma Finder 3.0 software by Thermo Fisher Scientific. A displays the deconvoluted masses of the free light chain (LC), the light chain dimer (LC/LC) and the fully formed cNISTmAb antibody, B provides a zoom-in on the intact antibody, revealing the masses of its main *N*-glycovariants.

Quality control mass spectrometry

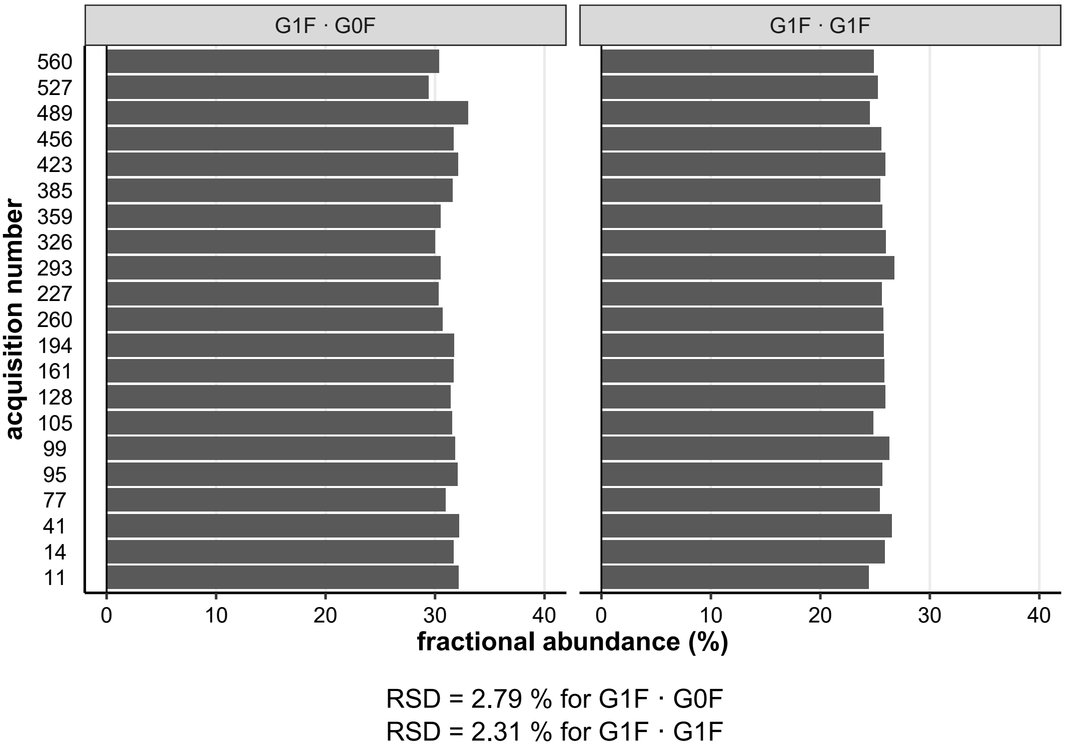

Supplementary Figure 8. Quality control sample NISTmAb (RM8671). Fractional abundances of major glycoforms of reference material NISTmAb (RM8671) across the duration of the acquisition with relative standard deviation (RSD). n = 21

Supplementary Table 7. Mass accuracy of QC measurements for the two most abundant glycoforms of NISTmAb (RM8671) evaluated by BioPharma Finder 3.0 software. n = 21

	G1F · G0F	G1F · G1F
Average Mass [Da]	148200.18	148361.89
SD	1.188	1.100
RSD %	0.000802	0.000741
Mass Deviation [ppm]	8.38	5.48
Mass Deviation [Da]	1.24	0.81

Clr-transformed N-glycan abundances

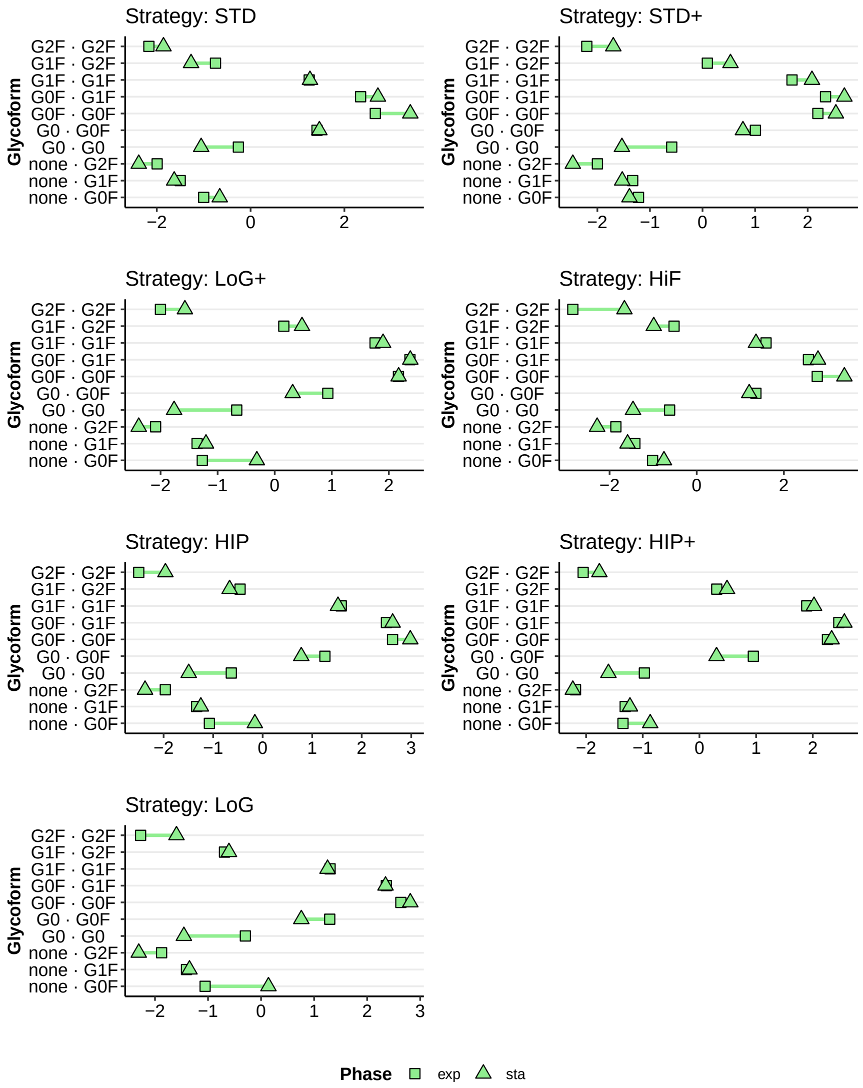

Supplementary Figure 9. Related to Figure 4. Timepoint-dependent changes in clr-transformed fractional abundances of N-glycans. Each point represents the mean clr value across biological replicates for a given glycoform and timepoint. Lightgreen lines connect means from two timepoints within the same glycoform, highlighting temporal trends. Note: For strategies STD, STD+, HiF, HIP, and HIP+, the stationary time point was collected at 264 hours, while for strategies LoG and Log+ it was 240 hours. The exponential time point for all strategies was 120 hours.

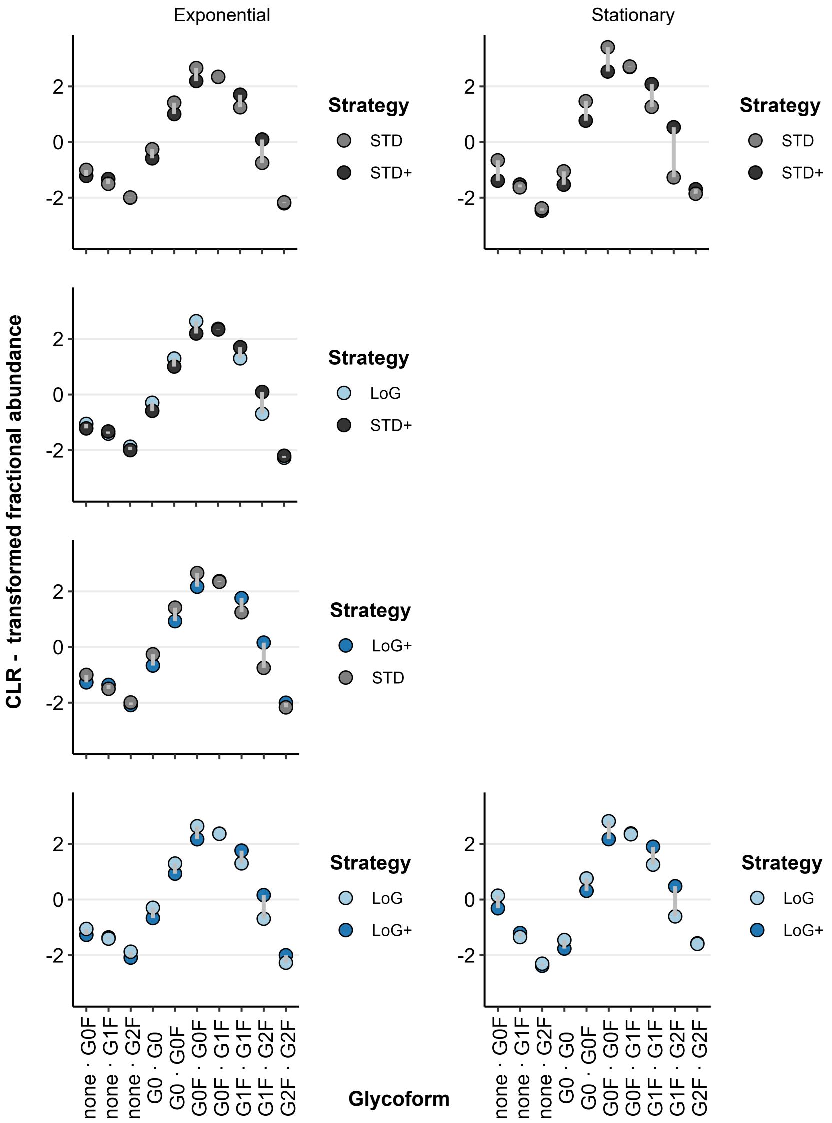

Supplementary Figure 10. Related to Figure 5A. Strategy-dependent changes in clr-transformed fractional abundances of *N*-glycans. Each point represents the mean clr value across biological replicates for a given glycoform and timepoint. Grey lines connect means from two feeding strategies within the same glycoform and timepoint, highlighting trends related to the feeding strategy.

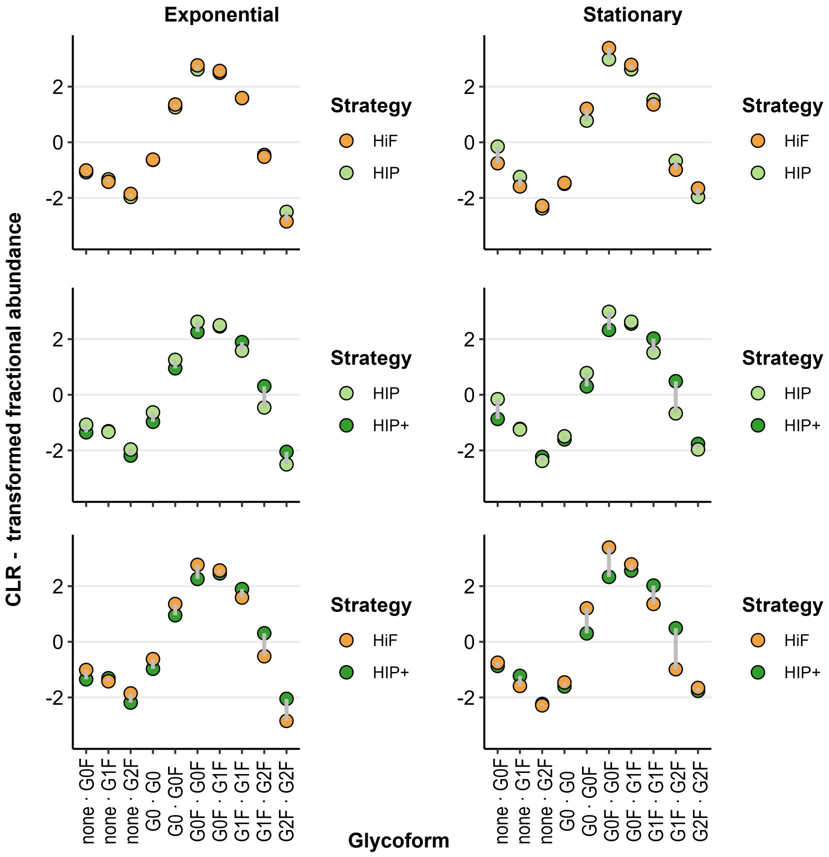

Supplementary Figure 11. Related to Figure 5B. Strategy-dependent changes in clr-transformed fractional abundances of *N*-glycans. Each point represents the mean clr value across biological replicates for a given glycoform and timepoint. Grey lines connect means from two feeding strategies within the same glycoform and timepoint, highlighting trends related to the feeding strategy.

Galactosylation index all pairwise statistics

Supplementary Table 8. Pairwise comparisons between feeding strategies for the galactosylation index. Reported are mean differences (diff), lower and upper 95% confidence intervals (lwr, upr), adjusted p-values (p adj), and significance levels (p <0.05 (*), p <0.01 (**), and p <0.001 (***)).

Group 1	Group 2	Time group	Diff	Lwr	Upr	p adj	Significance
HIP	HIP+	Exponential	-5.71	17.29	23.00	3.19E-02	*
LoG	LoG+	Exponential	-7.23	15.22	22.45	1.04E-06	***
HIP	HIP+	Stationary	-9.38	15.31	24.69	1.04E-06	***
STD	STD+	Exponential	-7.06	14.53	21.59	1.40E-07	***
LoG	LoG+	Stationary	-10.32	14.77	25.09	5.25E-08	***
STD	STD+	Stationary	-12.09	11.09	23.18	2.01E-09	***

Glycation index all Kruskall-Wallis results

Supplementary Table 9. Pairwise comparisons between feeding strategies for the glycation index. Reported are adjusted p-values (p adj) and significance levels (p <0.05 (*), p <0.01 (**), and p <0.001 (***)).

Group 1	Group 2	Time group	p adj	Significance
HIP+	LoG+	Stationary	9.23E-01	ns
STD	STD+	Stationary	9.23E-01	ns
HIP	HIP+	Stationary	9.11E-01	ns
HiF	HIP	Exponential	8.68E-01	ns
LoG	LoG+	Exponential	8.68E-01	ns
HIP	LoG+	Stationary	8.29E-01	ns
STD	STD+	Exponential	7.75E-01	ns
LoG	STD+	Stationary	7.38E-01	ns
HIP	HIP+	Exponential	7.21E-01	ns
LoG	STD	Stationary	6.99E-01	ns
HiF	HIP+	Exponential	6.25E-01	ns
HiF	HIP+	Stationary	5.81E-01	ns
HiF	LoG+	Stationary	5.81E-01	ns
HiF	STD	Stationary	5.81E-01	ns
HiF	STD+	Stationary	5.81E-01	ns
HiF	HIP	Stationary	5.10E-01	ns
HiF	STD+	Exponential	3.93E-01	ns
HiF	LoG	Stationary	3.34E-01	ns
HIP	STD+	Exponential	3.14E-01	ns
LoG+	STD	Exponential	3.14E-01	ns
HiF	STD	Exponential	2.64E-01	ns
LoG	STD	Exponential	2.62E-01	ns
HIP	STD	Exponential	2.22E-01	ns
LoG+	STD+	Exponential	2.15E-01	ns
HIP+	STD+	Exponential	1.87E-01	ns
LoG	STD+	Exponential	1.78E-01	ns
HIP	STD	Stationary	1.76E-01	ns
HIP	STD+	Stationary	1.76E-01	ns
HIP+	STD	Stationary	1.76E-01	ns
HIP+	STD+	Stationary	1.76E-01	ns
LoG+	STD	Stationary	1.76E-01	ns
LoG+	STD+	Stationary	1.76E-01	ns
HIP	LoG	Stationary	1.29E-01	ns
HIP+	LoG	Stationary	1.29E-01	ns
LoG	LoG+	Stationary	1.29E-01	ns
HIP+	STD	Exponential	1.06E-01	ns
HiF	LoG+	Exponential	3.77E-02	*
HiF	LoG	Exponential	2.68E-02	*
HIP	LoG+	Exponential	2.68E-02	*
HIP	LoG	Exponential	2.57E-02	*
HIP+	LoG	Exponential	1.16E-02	*
HIP+	LoG+	Exponential	1.16E-02	*
